# The interplay between the gut microbiota and metabolism during the third trimester of pregnancy

**DOI:** 10.3389/fmicb.2022.1059227

**Published:** 2022-12-07

**Authors:** Xinyuan Liang, Rongning Wang, Huijuan Luo, Yihong Liao, Xiaowen Chen, Xiaomin Xiao, Liping Li

**Affiliations:** ^1^Department of Obstetrics, The Second Clinical Medical College, Jinan University (Shenzhen People’s Hospital), Shenzhen, China; ^2^The First Affiliated Hospital, Jinan University, Guangzhou, China; ^3^Department of Cardiology, The Second Clinical Medical College, Jinan University (Shenzhen People’s Hospital), Shenzhen, China; ^4^Department of Obstetrics and Gynecology, The First Affiliated Hospital, Jinan University, Guangzhou, China

**Keywords:** gut microbiota, pregnancy, metabolism, correlation network, 16S rRNA gene sequencing

## Abstract

The gut microbiota undergoes dynamic changes during pregnancy. The gut microbial and metabolic networks observed in pregnant women have not been systematically analyzed. The primary purpose of this study was to explore the alterations in the gut microbiota and metabolism during late pregnancy and investigate the associations between the gut microbiota and metabolism. A total of thirty healthy pregnant women were followed from 30 to 32 weeks of gestation to full term. Fecal samples were collected for microbiome analysis and untargeted metabolomic analysis. The characteristics of the gut microbiota were evaluated by 16S ribosomal RNA gene sequencing of the V3-V4 regions. The plasma samples were used for untargeted metabolomic analysis with liquid chromatography–tandem mass spectrometry. The interplay between the gut microbiota and metabolism was analyzed further by bioinformatics approaches. We found that the relative abundances of *Sellimonas* and *Megamonas* were higher at full term, whereas that of *Proteobacteria* was lower. The correlation network of the gut microbiota tended to exhibit weaker connections from 32 weeks of gestation to the antepartum timepoint. Changes in the gut microbiota during late pregnancy were correlated with the absorbance and metabolism of microbiota-associated metabolites, such as fatty acids and free amino acids, thereby generating a unique metabolic system for the growth of the fetus. Decreasing the concentration of specific metabolites in plasma and increasing the levels of palmitic acid and 20-hydroxyarachidonic acid may enhance the transformation of a proinflammatory immune state as pregnancy progresses.

## Introduction

During human development, the microbiome adapted to dramatic variations that were reflective of the different body sites in which it was harbored. Within each site, the associated microbiome formed an enduring, stable, and distinctive microbial community. These unique biological entities respond to changes in their hosts, and the microbiome was involved in evolution (Rosenberg and Zilber-Rosenberg, 2018; Zmora et al., 2019; Suzuki and Ley, 2020). The intestinal compartment contained approximately 4 × 10^13^ bacteria that comprised the gut microbiota, which was roughly equal to the total number of human cells (Rosenberg and Zilber-Rosenberg, 2018). The number of microbial genes was approximately 100 times that of human genes (Gilbert et al., 2018; Rosenberg and Zilber-Rosenberg, 2018). The actual health state of humans can be estimated based on the structure and composition of the gut microbiota (Marchesi et al., 2016; Lavelle and Sokol, 2020). Due to the great diversity of microbial species and their contribution to host metabolism, the nervous system and other functions, the gut microbiota has become a novel biomarker for disease (DiGiulio et al., 2015; Gilbert et al., 2018).

The gut microbiota is influenced by a combination of genetic and environmental factors, and it changes dynamically during pregnancy (Koren et al., 2012; DiGiulio et al., 2015; Amir et al., 2020). The changes in the gut microbiota that occur over time are specific and vital for meeting the increasing energy and nutritional requirements of the fetus (Milani et al., 2017; Dicks et al., 2018). In addition, a series of complex metabolic alterations occur during the process of gestation, and are necessary for the successful maintenance of pregnancy (Lindsay et al., 2015; Liang et al., 2020). Although there is a consensus that the maternal gut microbiota and metabolism are crucial for the developing fetus and even neonates, our understanding of their correlations and involved mechanisms during the pregnancy remains incomplete.

Importantly, we have not yet clarified the variations in the gut microbiota, maternal metabolism and the microbial metabolome that occur during the third trimester of normal pregnancy or the potential influence on the onset of parturition. Moreover, the microbiota could function by producing metabolites that act as the intermediate compounds involved in the activities performed by both the host and microbiota (Marcobal et al., 2013). Therefore, an evaluation of the changes in the gut microbiota, the microbial metabolome and their correlations during normal pregnancy would be valuable. In this study, we collected the fecal samples and 4.0 ml peripheral venous blood samples from healthy pregnant women at 30–32 weeks of gestation and before parturition. The correlation networks among the gut microbiota, fecal metabolites and plasma metabolites were analyzed systematically. This study provides evidence for clinical management decisions and a more comprehensive understanding of the gut microbiota during pregnancy.

## Materials and methods

### Study design

Pregnant women with singleton pregnancy were recruited before 30 weeks of gestation with strict prenatal examination and monitoring in the outpatient department of the First Affiliated Hospital of Jinan University (Guangzhou, China). Participants with a history of gastrointestinal diseases and other diseases, especially recent infections, diabetes mellitus, autoimmune diseases and hyperthyroidism or perinatal complications were excluded. The participants had not taken any antimicrobial or immunological therapy within the previous 12 months. Probiotics or probiotic products were avoided. Women whose neonates had congenital diseases or low birth weight (less than 2,500 grams) during the study were also excluded. All participants were Han Chinese and from Southern China. Rice was a staple food consumed by the participants. Any changes in their diets were recorded during their antenatal examination. The study was approved by the institutional review board of the First Affiliated Hospital of Jinan University (2019–011). We obtained written informed consent from all the participants.

Thirty healthy pregnant women aged 22–30 years at 30–32 weeks of gestation were followed in the present study. Their progestational body mass index (BMI) was in the normal range. The participants shared comparable dietary habits and lifestyles. The dietary habits of all participants were relatively consistent during their pregnancy. We gathered antenatal information from the pregnant women by maintaining close contact with them. The samples obtained during 30–32 weeks of gestation were considered as the baseline (PF). The enrolled pregnant women provided their samples at full-term pregnancy (37–38 weeks of gestation) before parturition for later testing (PL). The average progestational BMI of the participants was 19.90 ± 1.46 (kg/m^2^). The weight gain of the women during pregnancy was 13.35 ± 4.42 kg. The duration of pregnancy was 277.00 ± 7.04 days. Five of the 30 pregnant women delivered by cesarean section. Detailed information on the pregnant women is provided in Supplementary Table S1. Among the neonates in our study, no severe birth complications or congenital diseases were observed.

### Sample collection and data acquisition

The fecal samples and approximately 4.0 ml peripheral venous blood samples were obtained from the participants in the fasting state upon enrollment and before parturition. The enrolled pregnant women were asked to abstain from enemas, vaginal douching or sexual intercourse 48 h prior to sampling. The participants were required to urinate and pay attention to cleaning before defecation to avoid introducing vaginal secretions or urine. They defecated on clean and sterile cotton pads. Trained professionals collected the samples with a uniform protocol for later testing. We took a spoonful (approximately 1.0 g) of internal substance and placed it vertically into the fecal collection tube. Peripheral venous blood samples were collected by professional nurses in EDTA anticoagulant tubes on the day of fecal sample collection. The samples were placed in a static position at room temperature for 20 min. Then, the supernatant was collected after centrifugation at 2000 rpm and 4°C for 10 min. All specimens were extracted aseptically. They were subsequently transferred to an ultralow temperature freezer with an icebox in 30 min.

We evaluated the features and composition of the gut microbiota by 16S rRNA gene sequencing of the V3-V4 regions. The 16S rRNA genes, which encodes the small subunit of rRNA in prokaryotes, have been widely used for taxonomic assignment and phylogenetic relationship determination in microbial studies (Sun et al., 2013; Church et al., 2020). Targeted sequencing of the V3 and V4 hypervariable regions is mainstream (Thijs et al., 2017; Fan et al., 2020) and it is considered as a suitable choice for the analysis of fecal samples (Abellan-Schneyder et al., 2021). Genomic DNA was extracted from the samples using the CTAB/SDS method as described previously (Wang et al., 2012). Universal primers and probe sets specifically designed to detect the 16S rDNA of prokaryotes and that of the domains Bacteria and Archaea were used (Yu et al., 2005). The traditional primer pair was recently shown to be biased against some bacteria. The primers were modified to reduce the biases and to achieve full coverage (Walters et al., 2016; Thijs et al., 2017). The primers used for amplification in our study were modified 341F/806R (Liu et al., 2020). Detailed sequencing information is provided in the Supplementary material. The sequences were assigned to operational taxonomic units (OTUs) with 97% similarity. Some commonly used methods of microbial analysis were performed (Gloor et al., 2017). The OTUs were assigned a taxonomy based on the SILVA 16S rRNA gene reference database (Pruesse et al., 2007).^1^

The fecal and plasma samples were collected for untargeted metabolomics detection by liquid chromatography–tandem mass spectrometry (LC–MS/MS) analysis (Supplementary material). The raw data were transferred into mzXML format by ProteoWizard (Chambers et al., 2012). Ion peaks of metabolites from all samples and QC samples were extracted using XCMS software (Smith et al., 2006) and analyzed with Pareto scaling. The ionization source of the QE platform was ESI in the positive ion mode and negative ion modes. The structure of the metabolite was determined by precise mass matching (<25 ppm) and secondary spectrum diagram matching to retrieve the self-built database. Unknown metabolites were represented with their ID (generated from the molecular formulae).

### Statistical analyses

The clinical characteristics were analyzed by using SPSS (Version 26.0, Chicago, IL, United States). The data are presented as the mean ± standard deviation (SD). The microbial analysis data were plotted with R (version 4.0.3)^2^ and GraphPad Prism (version 8.4.3, California, United States) software. Gephi (version 0.9.2)^3^ was used to generate the relational network. Differential analysis was performed using a two-tailed paired *t* test for measurement data. Taxa with the same OTU type were clustered at the phylum, class, order, family, and genus levels. Alpha diversity was analyzed by the Shannon and Simpson indexes. Beta diversity was estimated by computing the unweighted UniFrac and weighted UniFrac algorithm distances. Linear discriminant analysis effect size (LEfSe) was performed, and the cladogram was drawn with default parameters (LDA score > 3.0). LEfSe is an algorithm for high-dimensional biomarker discovery and interpretation. It was used to identify differential features with statistical significance, biological consistency and effect relevance (Segata et al., 2011). MetaStat analysis was subsequently used to compare group differences. The results were adjusted by using the Benjamini–Hochberg procedure with the critical false discovery rate (FDR) set to 0.05. The correlation analysis was performed with data filtered at the genus level, covering only those relative abundances presented in the top 100 of the samples in each group. The edges were estimated by Spearman’s confidence index [abs(r) > 0.6]. After relative standard deviation denoising and standard internal normalization, the final metabolite dataset was imported to SIMCA-P 14.1 (Umetrics, Umea, Sweden) for further analysis. Supervised orthogonal projections to latent structures-discriminant analysis (OPLS-DA) was performed based on principal component analysis. The validity of the model was evaluated using R^2^ (model’s interpretability to variables) and Q^2^ (predictability of the model) values obtained from seven-fold cross-validation. The variable importance in the projection (VIP) of the first principal component of the OPLS-DA was calculated to identify the important metabolites. The metabolites with VIP >1.0 were identified as contributing variables to the classifications. Kyoto Encyclopedia of Genes and Genomes (KEGG)^4^ and MetaboAnalyst^5^ were used for pathway-enrichment analyses. The origin and function analysis of metabolites was performed using MetOrigin.^6^ The correlations between the gut microbiota and metabolites were assessed. Results with a *p*-value below 0.05 were considered statistically significant.

## Results

### The altered composition of the gut microbiota

A total of 60 fecal samples from the pregnant women obtained at two phases (30–32 weeks of gestation: PFF; term gestation: PLF) were analyzed by 16S rRNA gene sequencing. All OTUs were detected under sufficient sequencing depth (Supplementary Figure S1A). The average number of effective tags after quality and chimera checking was 60,790 per sample. The average length obtained was 414 bp. A total of 5,043 OTUs at the 97% similarity level were determined. There were 3,579 and 3,637 OTUs classified in the PFF and PLF groups, respectively. They had 2,173 common OTUs (Supplementary Figure S1B). Alpha diversity was estimated by the Shannon index (PFF: 5.39 ± 0.90; PLF: 5.54 ± 0.57) and Simpson index (PFF: 0.91 ± 0.12; PLF: 0.93 ± 0.03). The analysis of alpha diversity indicated that there was no difference between the two stages (Supplementary Figure S1C). Moreover, the composition of the gut microbiota was assessed by the principal coordinate analysis (PCoA). We observed an obvious variation among the groups (*p* < 0.01; Supplementary Figure S1D). We conducted compositional analysis with a variance-based compositional principal component (PCA) biplot. The relationship between the inter-variance and sample distance was observed from different perspectives. No obvious separation was found between the two groups (Supplementary Figure S2).

We then further analyzed the alterations in the gut microbial composition at the phylum and genus levels. *Firmicutes*, *Bacteroidota*, *Proteobacteria*, and *Actinobacteriota* represented more than 95% of the classifications at the phylum level. The relative abundance of *Proteobacteria* was notably higher at baseline (PFF, 14.14%) than at term gestation (PLF, 4.05%; Supplementary Figure S3A). At the genus level, the relative abundances of *Faecalibacterium*, *Bacteroides*, and *Bifidobacterium* showed a remarkable upward trend at the full-term pregnancy timepoint, whereas the relative abundance of *Escherichia-Shigella* (PFF: 11.68%, PLF: 2.58%) showed a downwards trend among the top ten abundant genera (Supplementary Figure S3B).

**Figure 1:**
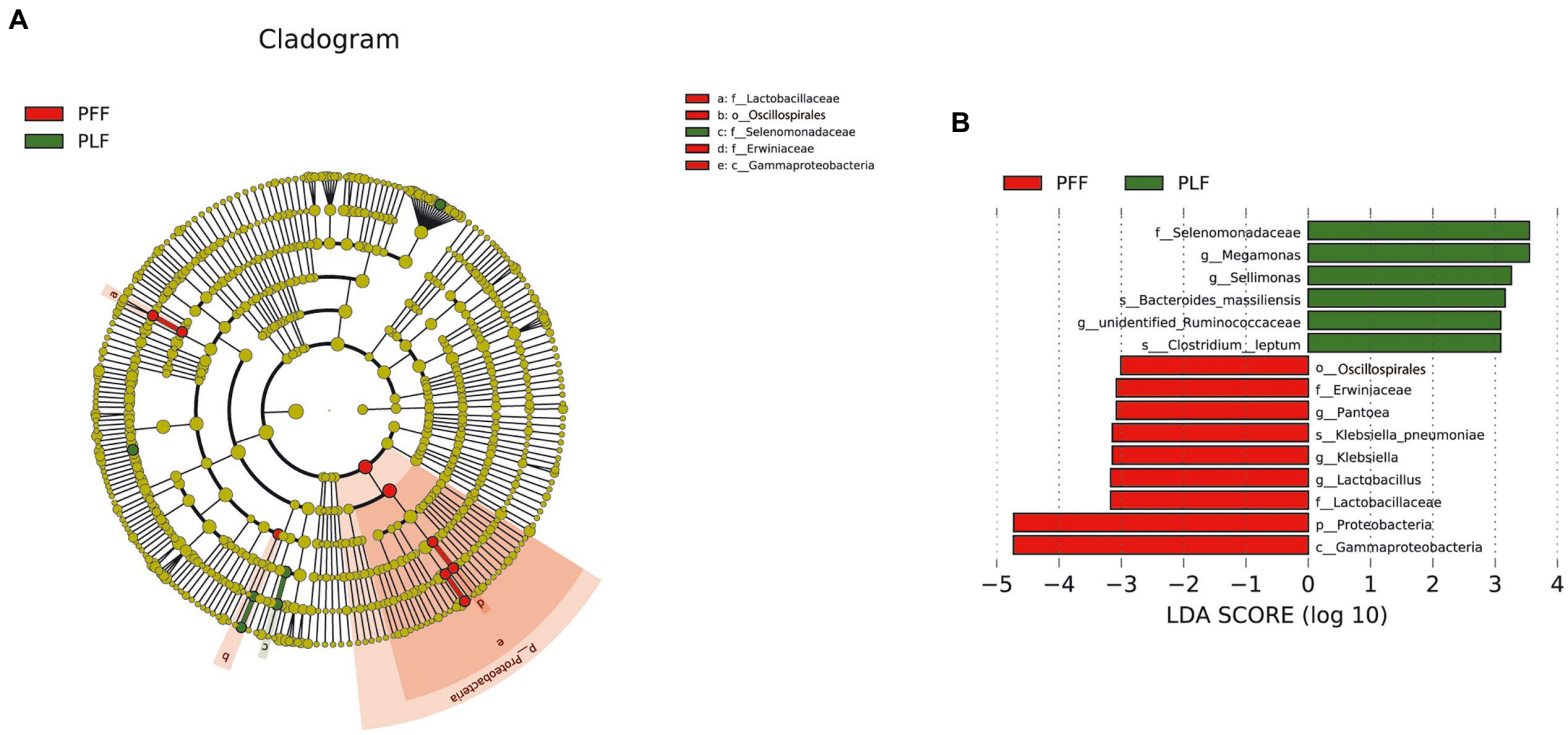
The identified biomarkers of gut microbiota composition by LEfSe analysis. **(A,B)** The LEfSe plot is presented with a threshold LDA score of 3.0. There were nine identified biomarkers (six identified genera) that differed between the PFF and PLF groups. PFF, pregnant women at 30–32 weeks of gestation. PLF, pregnant women at full term.

Furthermore, the difference in the composition of the gut microbiota was measured with LEfSe. Six genera were identified (Figures 1A,B). The relative abundances of *Megamonas* (1.07%) and *Sellimonas* (0.52%) were markedly higher at full term than at the baseline. In addition, a marked decrease in the relative abundances of *Lactobacillus* (0.15%), *Pantoea* (0.13%), and *Klebsiella* (0.10%) was also observed (Table 1). Interestingly, *Megamonas* comprised a low proportion in the composition of the microbial community at 30–32 weeks of gestation, but was highly abundant in full-term pregnancy, indicating the potential importance of this genus (Table 1).

**Table 1:** The differential microbiota at different levels of biological classification with LEfSe analysis and MetaStat analysis.

Phylum	Class	Order	Family	Genus	PFF (*n* = 30, %)	PLF (*n* = 30, %)	*p*-value
Firmicutes	Clostridia	Oscillospirales			0.39 ± 1.22	0.39 ± 0.54	0.999
		Oscillospirales	Ruminococcaceae	unidentified_Ruminococcaceae	0.05 ± 0.05	0.26 ± 0.55	0.001ª
		Lachnospirales	Lachnospiraceae	Sellimonas	0.16 ± 0.18	0.52 ± 1.08	0.016
	Negativicutes	Veillonellales-Selenomonadales	Selenomonadaceae	Megamonas	0.15 ± 0.49	1.07 ± 4.17	0.142
	Bacilli	Lactobacillales	Lactobacillaceae	Lactobacillus	0.37 ± 1.35	0.15 ± 0.42	0.551
							
Proteobacteria*^#^*					14.14 ± 20.11	4.05 ± 3.09	0.006ª
	Gammaproteobacteria*^#^*				13.91 ± 20.10	3.78 ± 3.02	0.004ª
		Enterobacterales	Erwiniaceae	Pantoea	0.39 ± 0.63	0.13 ± 0.11	0.005ª
			Enterobacteriaceae	Klebsiella	0.40 ± 0.60	0.10 ± 0.09	0.002ª

^#^LDA score > 4.0; ªq-value < 0.05.

### The correlation network of the gut microbiota during the late pregnancy

**Figure 2:**
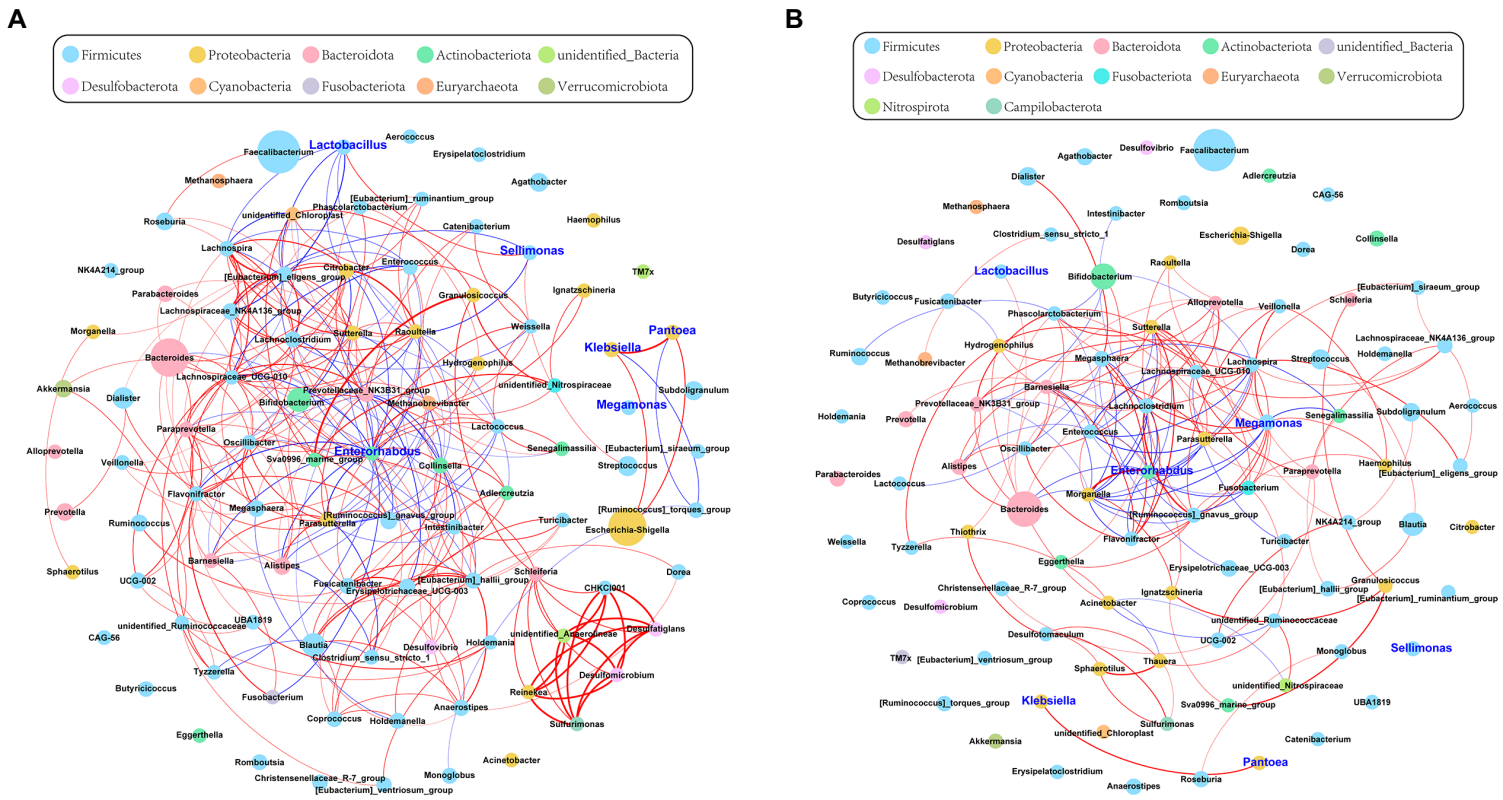
The network of gut microbiota during late pregnancy (based at the genus level). Each node was annotated with the name. The size of the nodes indicates their relative abundance in each group. The color of each node represents the phylum. The red and blue lines represent the positive and negative relationships between each node, respectively. **(A)** The microbial community was complex and balanced at baseline (30–32 weeks of gestation). The genera of *Proteobacteria* (orange) were pointedly active. **(B)** As gestational age progressed, the whole network tended to be looser. The relative abundance of *Megamonas* (sky blue) and its correlations with other genera were significantly increased in the full-term pregnancy. *Enterorhabdus* (green) held a central position during late pregnancy. However, the microbial connections were weak and varied. The absolute value of Spearman rank correlation coefficient was >0.6, and *P* was <0.05.

To investigate the correlations among the gut microbiota, we computed the correlation coefficients and drew correlation networks by igraph (R package) and Gephi (version 0.9.2) with a cutoff of 0.6 and *p* < 0.05. Each node was annotated at the phylum level. The size of the nodes represents their relative abundance. The red and blue lines represent the positive and negative relationships between each node, respectively. The tightness and complexity of the microbial correlation network decreased from the baseline to full term (Figures 2A,B). The networks shown by modularity classification are provided (Supplementary Figures S4A,B). The gut microbiota responded sensitively to gestational age and their correlations decreased gradually.

To further determine the effect of gestational age on the changes in the microbial correlation network, we calculated the index of the correlation network at two stages. The network diameters at baseline and full term were 8 and 10, respectively. The average degree and clustering coefficient showed that the correlations of microbes decreased sharply. The microbial correlation network was less organized at term gestation (Table 2). As gestation proceeded, a less complex microbial network was observed. Some differential genera were identified by LEfSe, and they might have played a key role in the microbial correlation network. At baseline, the relative abundance of *Lactobacillus* was negatively correlated with the abundances of five genera, and was clustered to be positively correlated with only the abundance of *Sellimonas*. However, the influence of *Lactobacillus* was reduced and changed with time. The relationship between the Lactobacillus and *Sellimonas* disappeared. In addition, the social behavior of *Sellimonas* was completely changed. Of note, some taxa with low abundance at 30–32 weeks of gestation were enriched significantly during full-term pregnancy. The increased abundance of *Megamonas* was attributed to the complexity of the microbial network at the full term. The relative abundance of *Megamonas* was positively associated with the abundances of nine genera and negatively correlated with the abundances of three genera but not at baseline (Table 3). It seemed that *Megamonas* was the most susceptible to changes in the gut microbiota. The positive correlation between the abundances of *Pantoea* and *Klebsiella* was reserved (Figures 2A,B). *Enterorhabdus* was markedly active during late pregnancy (Supplementary Figures S4A,B). The above results indicated that the correlation network of the gut microbiota during late pregnancy is extremely complex and undergoes dynamic changes.

**Table 2:** The networking of gut microbiota in different phase.

	PFF	PLF
Generic relationships	296	157
Network diameter (ND)	8	10
Modularity (MD)	0.489	0.451
Clustering coefficient (CC)	0.605	0.504
Graph density (GD)	0.064	0.034
Average degree (AD)	4.241	3.237
Average path length (APL)	3.073	3.513

**Table 3:** The changes of correlation in the network of gut microbiota.

Phylum	Genus	Correlations	PFF	PLF
Actinobacteriota	Enterorhabdus	+	8	3
		−	22	12
Firmicutes	Lactobacillus	+	1	1
		−	5	0
	Sellimonas	+	2	0
		−	2	0
	Megamonas	+	0	9
		−	0	3
	unidentified_Ruminococcaceae	+	4	3
		−	0	1
Proteobacteria	Klebsiella	+	2	1
		−	1	0
	Pantoea	+	2	1
		−	1	0

The symbols present Spearman’s correlation co-efficiency: (+) indicates positive relationship, (−) indicates negative relationship.

### Identification of differential metabolites in fecal and plasma samples

**Figure 3:**
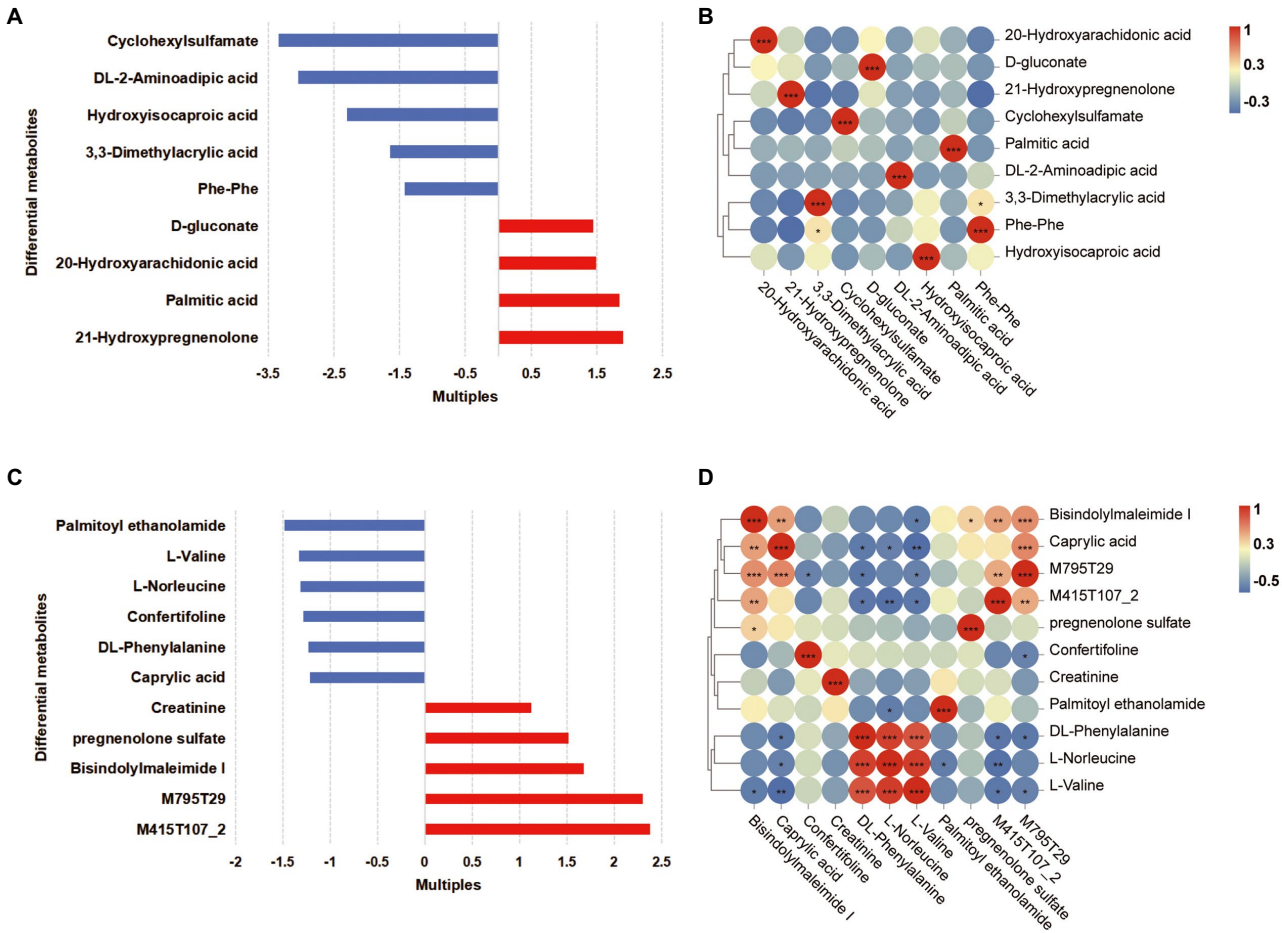
Differential metabolites in the fecal and plasma samples of women in full-term pregnancy. **(A)** The fold changes of differential metabolites in the fecal samples. Each bar colored by the red and blue indicates increase and reduction, respectively. **(B)** The correlation heatmap of fecal metabolites. **(C)** The fold changes of differential metabolites in the plasma samples. Each bar colored by the red and blue indicates increase and reduction, respectively. **(D)** The correlation heatmap of plasma metabolites. *p < 0.05; **0.001 ≤ *p* < 0.01; ***p < 0.001.

The OPLS-DA plots of the metabolomics data showed the separation of pregnant women at different phases (plasma samples: PFB vs. PLB; fecal samples: PFF vs. PLF; Supplementary Figure S5A). A total of 620 fecal metabolites and 311 plasma metabolites were identified. The structural identification of metabolites was achieved by using a self-built database (Supplementary Figures S5B,C). Differential metabolites were identified using selection criteria of VIP >1.0 and *p* value <0.05 between groups in the fecal and plasma samples, respectively. The identified differential metabolites may be core metabolites involved in late pregnancy. The classifications of differential metabolites and the fold changes are shown in Tables 4, 5. We identified the nine differential metabolites in the fecal samples. According to the variable importance of differential metabolites, the pivotal metabolites were ranked in the following order: cyclohexylsulfamate, 3,3-dimethylacrylic acid, hydroxyisocaproic acid, 20-hydroxyarachidonic acid and phenylalanylphenylalanine (Phe-Phe). The levels of lipids and lipid-like molecules, such as long-chain fatty acids and 21-hydroxysteroids, in the fecal samples obtained from pregnant women showed an upward trend from baseline to full term, whereas the levels of amino acids and dipeptides showed a downward trend. 20-Hydroxyarachidonic acid and palmitic acid were enriched at the time of full-term pregnancy (Table 4; Figure 3A). The concentration of 3,3-dimethylacrylic acid had a positive relation with the level of phenylalanylphenylalanine (*r* = 0.407; Figure 3B). The enriched pathways identified based on fecal samples are provided in Supplementary Figure S6A.

**Table 4:** Differential metabolites in fecal samples of PLF and PFF.

Ionization mode	Name	Super class	Direct parent	HMDB	KEGG	VIP	Fold change	*p*-value
Positive (+)	3,3-Dimethylacrylic acid	Lipids and lipid-like molecules	Methyl-branched fatty acids	HMDB0000509	–	5.525	0.609	0.040*
	20-Hydroxyarachidonic acid	Lipids and lipid-like molecules	Long-chain fatty acids	HMDB0005998	C14748	2.53	1.491	0.031*
	Phe-Phe	Organic acids and derivatives	Dipeptides	HMDB0013302	–	2.133	0.704	0.0496*
	DL-2-Aminoadipic acid	Organic acids and derivatives	L-alpha-amino acids	HMDB0000510	C00956	1.761	0.329	0.049*
	Palmitic acid	Lipids and lipid-like molecules	Long-chain fatty acids	HMDB0000220	C00249	1.447	1.839	0.013*
Negative (−)	Cyclohexylsulfamate	Organic acids and derivatives	Cyclamates	HMDB0031340	C02824	57.093	0.299	0.034*
	Hydroxyisocaproic acid	Lipids and lipid-like molecules	Hydroxy fatty acids	HMDB0000746	-	4.361	0.435	0.002**
	21-Hydroxypregnenolone	Lipids and lipid-like molecules	21-hydroxysteroids	HMDB0004026	C05485	1.443	1.896	0.044*
	D-gluconate	Organic oxygen compounds	Sugar acids and derivatives	HMDB0000625	C00257	1.056	1.44	0.0498*

PFF, the fecal samples of pregnant women at 30–32 weeks of gestation; PLF, the fecal samples of pregnant women at full term. *p < 0.05; **0.001 ≤ *p* < 0.01; –: Undefined.

Furthermore, eleven differential metabolites were identified in the plasma samples. The pivotal plasma metabolites were as follows: bisindolylmaleimide I, palmitoyl ethanolamide, confertifoline, M795T29 and creatinine. There were two differential metabolites, which were M795T29 and M415T107_2, that had molecular structures similar to the structures of sunitinib and ramipril, respectively. These metabolites were classified as indoles and derivatives, and their concentration increased gradually as pregnancy progressed. We ensured that the participants did not take any medication during pregnancy. These substances might be generated biologically as a result of pregnancy. We found that there was a significant decrease in amino acids and derivatives and caprylic acid in plasma samples (Table 5, Figure 3C). At the time of full-term pregnancy, the plasma metabolites L-norleucine, DL-phenylalanine and L-valine were positively correlated (*r* > 0.5, *p* < 0.05). Caprylic acid was a medium-chain fatty acids and its level showed positive correlations with the levels of bisindolylmaleimide I and M795T29. The correlations among the levels of plasma metabolites are provided in Figure 3D and Supplementary Table S3. Two metabolites’ pathways identified using the plasma samples were found to be significantly involved (*p* < 0.05, impact value>0.1; Supplementary Figure S6B). These included phenylalanine, tyrosine and tryptophan biosynthesis and phenylalanine metabolism.

**Table 5:** Differential metabolites in plasma samples of PLB and PFB.

Ionization mode	Name	Super class	Direct parent	HMDB	KEGG	VIP	Fold change	*p*-value
Positive (+)	Palmitoyl ethanolamide	Organic acids and derivatives	Carboximidic acids	HMDB0002100	C16512	4.173	0.673	0.014*
	Creatinine	Organic acids and derivatives	Alpha amino acids and derivatives	HMDB0000562	C00791	2.424	1.124	0.017*
	L-Norleucine	Organic acids and derivatives	L-alpha-amino acids	HMDB0001645	C01933	2.4	0.763	0.016*
	DL-Phenylalanine	Organic acids and derivatives	Phenylalanine and derivatives	HMDB0000159	C00079	1.564	0.812	0.022*
	L-Valine	Organic acids and derivatives	Valine and derivatives	HMDB0000883	C00183	1.39	0.753	0.003**
Negative (−)	Bisindolylmaleimide I	Organoheterocyclic compounds	N-alkylindoles	HMDB0249269	C11238	4.367	1.678	<0.001***
	Confertifoline	–	–	–	–	3.807	0.779	0.001**
	M795T29	–	–	–	–	2.744	2.302	0.010*
	Caprylic acid	Lipids and lipid-like molecules	Medium-chain fatty acids	HMDB0000482	C06423	1.539	0.822	0.005**
	Pregnenolone sulfate	Lipids and lipid-like molecules	Sulfated steroids	HMDB0000774	–	1.199	1.516	0.002**
	M415T107_2	–	–	–	–	1.022	2.376	0.019*

PFB, the plasma samples of pregnant women at 30–32 weeks of gestation; PLB, the plasma samples of pregnant women at full term. *p < 0.05; **0.001 ≤ *p* < 0.01; ***p < 0.001. –: Undefined.

### The relationships among the gut microbiota, fecal metabolites and plasma metabolites

**Figure 4:**
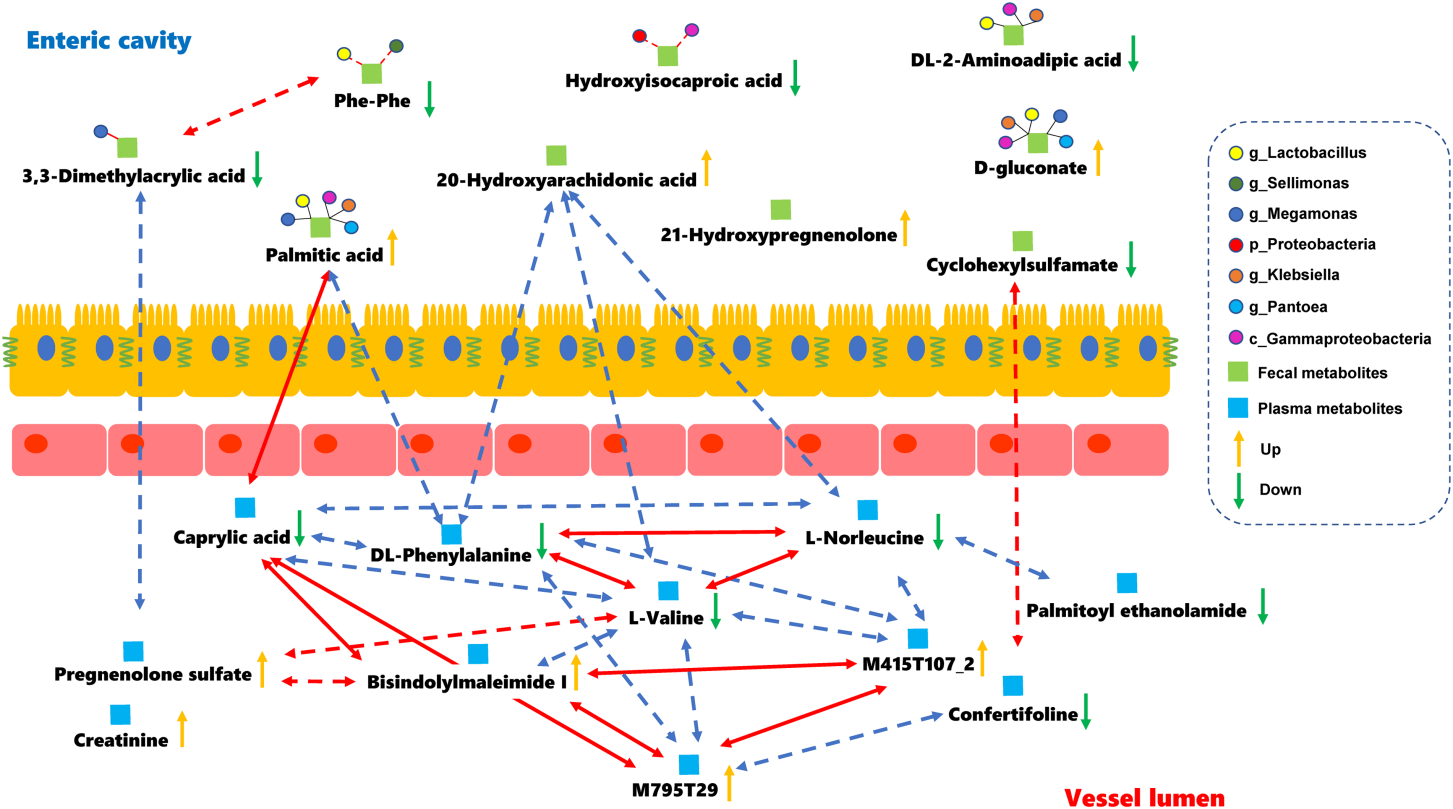
Microbes-fecal metabolites-plasma metabolites network in the full-term pregnancy. The red and blue lines represent the positive and negative relationships, respectively. The absolute value of the correlation coefficient was equal to or greater than 0.5. The dark lines indicate that there were some biological relationships between the metabolites and identified gut microbes. Dotted lines represent that the absolute value of the *Pearson* correlation coefficient was greater than 0.3 but less than 0.5 with statistical significance.

The relationships between the gut microbiota and differential metabolites during full-term pregnancy were assessed by Pearson correlation analysis. We found that the relative abundance of *Proteobacteria* was positively correlated with the level of hydroxyisocaproic acid, whereas the abundance of identified genera of *Firmicutes* was correlated with the levels of 3,3-dimethylacrylic acid and phenylalanylphenylalanine (Supplementary Figure S7; Supplementary Table S4). The concentration of caprylic acid in plasma was positively correlated with the level of palmitic acid in fecal samples, indicating that these core metabolites might be important mediators involved in full-term pregnancy. Additionally, the level of 20-hydroxyarachidonic acid in the fecal samples was negatively correlated with the plasma metabolites L-norleucine, DL-phenylalanine and L-valine (Supplementary Table S5). The plasma metabolites-M795T29 (a substance similar to sunitinib in structure) and bisindolylmaleimide I, which are the derivatives of indoles, could be potential regulators that mediate interactions between the digestive tract and circulation (Figure 4). Furthermore, we distinguished the differential metabolites originating from the microbial community or host by MetOrigin. The 20-hydroxyarachidonic acid in the fecal samples originated from metabolic processes occurring in the host. The other metabolites might have been synthesized endogenously or concentrated as gestational age progressed. Hydroxyisocaproic acid is a type of fatty acid produced by the gut microbiota. The level of this metabolite showed a weak and positive correlation with the abundance of *Proteobacteria* in our study. The detailed information regarding the source of the differential metabolites is listed in Supplementary Table S6. We focused on the fecal metabolites involved in metabolic processes shared by the host and microbiota to explore their relationships. DL-2-aminoadipic acid, palmitic acid and D-gluconate are commonly considered to be metabolized by the host and gut microbiota. The potential correlations and biological relationships among the metabolites and identified gut microbes were obtained from MetOrigin. *Gammaproteobacteria, Lactobacillus*, and *Klebsiella* played important roles in the metabolic processes common to the host and the gut microbiota. The pentose phosphate pathway (PPP) was statistically and biologically enriched at the time of full-term pregnancy (Supplementary Figure S6A; Supplementary Table S7). Details regarding the microbe-metabolite network are presented in Figure 4.

## Discussion

The actual variations in the gut microbiota that occur during normal pregnancy remain unknown. Accumulating evidence has suggested that abnormal variations in the gut microbiota are closely related to perinatal complications, such as preterm birth (DiGiulio et al., 2015), gestational diabetes mellitus (GDM; Ponzo et al., 2019; Hasain et al., 2020) and other complications (Lv et al., 2019). The transmission of microbiota from mother to child was a key determinant of the health of the child (DiGiulio et al., 2015; Younes et al., 2018). The gut microbiota plays an important role in maternal physiological activities. The interactions between the gut microbiota and host metabolism during pregnancy have not yet been elucidated. Thus, clarifying the changes that occur in the gut microbiota and identifying the key bacteria that can serve as biological markers during normal late pregnancy are valuable avenues of investigation. This study characterized the changes and correlations in the gut microbiota that occurred at various time-points. It also provided a corresponding basis for the gut microbiota as a target of clinical intervention or indicator for treatments targeting metabolism.

The alpha diversity indexes showed no difference between the two groups. As pregnancy progressed, the beta diversity of the gut microbiota increased significantly. This result was consistent with the findings of a previous study (Koren et al., 2012). There was a remarkable increase in the β-diversity of the gut microbiota with gestational age. The results also suggested that the composition and structure of the microbial community changed dynamically during pregnancy. However, some scholars have pointed out that the diversity of the gut microbiota is unrelated to gestational age during pregnancy and that the microbial structure remains stable (DiGiulio et al., 2015). The variations in β-diversity might be the result of hormonal changes or immune tolerance during pregnancy.

In the current study, the relative abundance of *Proteobacteria* was approximately three-fold higher than that in the full-term stage at the phylum level. At the genus level, the relative abundance of *Lactobacillus, Pantoea*, and *Klebsiella* showed a downward trend, whereas *Sellimonas* and *Megamonas* of *Firmicutes* increased significantly by the time of full-term pregnancy. *Lactobacillus* can colonize the vagina and gastrointestinal tract and provides substantial benefits from its appropriate colonization (Mu et al., 2018; Barrientos-Durán et al., 2020). Thus, it is often supplemented in the diet as an important component of probiotics (Liévin-Le Moal and Servin, 2014; Dobrut et al., 2018). *Lactobacillus* is a gram-positive facultative anaerobe (Mu et al., 2018). The time-dependent decrease in *Lactobacillus* abundance during pregnancy might lead to the increase in the levels of some fastidious anaerobes. *Lactobacillus* can take part in food fermentation and produce related metabolites, including lactic acid, bacteriocins, non-bacteriocins, and nonprotein molecules with direct bactericidal effects (Liévin-Le Moal and Servin, 2014). The relative abundance of rectal *Lactobacillus* in healthy pregnant women in Poland has been reported to decrease significantly during late pregnancy (Dobrut et al., 2018), which was basically consistent with our results. Interestingly, the abundances of *Lactobacillus* species obtained from gut samples that have a certain degree of homology decreased with gestational age, while the vaginal abundance of *Lactobacillus* was found to be increased in a metagenomic analysis (Goltsman et al., 2018). The decrease in *Lactobacillus* and its transformation during late pregnancy should be investigated and confirmed in more clinical experiments. *Pantoea* is a gram-negative and facultative anaerobe. It belongs to the *Enterobacteriaceae* family and *Proteobacteria*, which are widely distributed and can be isolated from water, soil, humans, animals and plants (Wang et al., 2017; Tambong, 2019). *Pantoea* has been studied as an opportunistic pathogen and is often associated with occupational exposure and community-acquired or hospital-acquired infections (Mani and Nair, 2021). In addition, it is closely related to premature rupture of membranes and preterm birth (Tiwari and Beriha, 2015). Similar to *Pantoea*, *Klebsiella* is ubiquitous in the environment. It can colonize the skin, or the gastrointestinal and respiratory tracts of healthy people (Patro and Rathinavelan, 2019; Herridge et al., 2020). However, it can infect and spread among neonates, elderly individuals and immunocompromised patients (Bengoechea and Sa Pessoa, 2019). More importantly, *Klebsiella* is associated with the occurrence of urinary tract infections during pregnancy (Lee et al., 2019) and gestational diabetes mellitus (Kuang et al., 2017). It is an important pathogen causing recurrent abortion (Omwandho et al., 2006), premature rupture of membranes and neonatal infection (Bonasoni et al., 2021). In our study, all the participants were healthy pregnant women in the third trimester of pregnancy. The differences in the abundances of *Pantoea*, *Klebsiella* or even *Proteobacteria* from 30 to 32 weeks of gestation to full term might be an adaptive change that occurs during pregnancy. With the progress of pregnancy, the relative abunance of *Sellimonas* and *Megamonas* tended to increase. *Sellimonas* is an anaerobic and gram-positive microbe in the family *Lachnospiraceae* of *Firmicutes* (Seo et al., 2016; Muñoz et al., 2020). The relative abundance of *Sellimonas* increases gradually with the improvement of intestinal barrier function, such as during the recovery of the intestinal barrier after chemotherapy for colorectal cancer or therapeutic splenectomy for liver cirrhosis. *Sellimonas* might be a candidate biomarker for intestinal homeostasis (Muñoz et al., 2020). Acetic acid could be produced by *Sellimonas* during glucose fermentation (Seo et al., 2016). A higher abundance of *Megamonas* was observed at full term in our study. This enriched genus might play a vital role in maternal metabolism. Its actual impact on pregnancy remains controversial. *Megamonas* could act as a beneficial microbe. However, the overgrowth of *Megamonas* could be involved in the occurrence of gestational anemia (Wei et al., 2021). Increased *Megamonas* abundance has been demonstrated to be associated with major depressive disorder (Wang et al., 2021). The relative abundance of *Megamonas* showed a positive correlation with BMI during pregnancy (Zhan et al., 2021; Abdullah et al., 2022). There was a downward trend of *Megamonas* in intrahepatic cholestasis of pregnancy (ICP) patients (Zhan et al., 2021), while the trend in GDM patients was exactly the opposite (Abdullah et al., 2022). The change in *Megamonas* might be associated with metabolite alterations that occur during late pregnancy.

Additionally, it is worth mentioning that the microbial network observed during the antepartum period became less complex and the correlations were reduced. The correlations among the microbes changed in the third trimester of pregnancy. The variation in the microbial structure should be considered holistically rather than based on specific bacterial species. This result indicated that the correlations among the microbes associated with the microbial community may be profoundly affected by metabolism in the third trimester of pregnancy. The regulatory role of the microbial network during pregnancy has not yet been explicitly elucidated in the literature from a biological perspective.

The intestinal microbial ecosystem takes part in the synthesis of vitamins and metabolism of the host for maintaining the intestinal and immune homeostasis (Kasubuchi et al., 2015). Metabolites are essential in the modification of the microbial signatures. They can serve as intermediaries for microbial functional activities and the interactions with the host (Marcobal et al., 2013; Xu et al., 2020). In this study, some metabolites with significant changes in their levels in the fecal and plasma samples obtained from pregnant women at full term were identified. The differential metabolites consisted mainly of lipids and lipid-like molecules, or organic acids and derivatives. The levels of microbiota-generated metabolite, hydroxyisocaproic acid tended to decrease, while 21-hydroxypregnenolone excreted by the host in the fecal samples tended to increase with gestational age. Hydroxyisocaproic acid is an end product of leucine metabolism (Liebich and Först, 1984; Sakko et al., 2012). Hydroxyisocaproic acid produced from the fermentation of *Lactobacillus* has shown potential as a topical antibiotic, and it also exerts anti-inflammatory properties (Sakko et al., 2021). Our results suggested that the reduction in the level of hydroxyisocaproic acid during full-term pregnancy was associated with a decrease in the abundance of *Lactobacillus.* 21-hydroxypregnenolone could originate from the fetuses and its high concentration played an essential role in deoxycorticosterone synthesis during pregnancy (Guerami et al., 1988). It was reported that 21-hydroxypregnenolone could be a biomarker of hypothalamic–pituitary–adrenal (HPA) axis abnormalities and immune dysfunction. This molecule has been found to help improve memory, prevent fatigue, and relieve stress (Shao et al., 2017). In our study, the increase in 21-hydroxypregnenolone was the obvious manifestation of pregnancy progression. In addition, an increase in the levels of long-chain fatty acids (palmitic acid and 20-hydroxyarachidonic acid) was observed in our study. Palmitic acid is a straight chain saturated fatty acid that can be absorbed from food or generated from the endogenous synthesis of some other fatty acids, carbohydrates and amino acids (Innis, 2016). It was reported that palmitic acid may have adverse impacts on conception and preimplantation embryo development (Innis, 2016; Rampersaud et al., 2020; Schindler et al., 2020). Interestingly, fetuses were found to accumulate palmitic acid during the last trimester of gestation. The accumulation of palmitic acid during late pregnancy is essential for the growth of membrane lipids, protein palmitoylation and the production of palmitoylated signaling molecules (Innis, 2016). Previous studies (Aparicio et al., 2021; Li et al., 2021) also reported that palmitic acid increased significantly in the third trimester of normal pregnancy, which was consistent with the findings of our study. Nevertheless, the abnormal accumulation of palmitic acid might give rise to inflammation at the maternal–placental interface, which resulted in pregnancy complications (such as preeclampsia, GDM and unexplained recurrent pregnancy loss; Nandakumaran et al., 2000; Rampersaud et al., 2020; Sano et al., 2020).

20-Hydroxyarachidonic acid (20-HETE) is a key factor in the development of endocrine metabolic diseases and it was one of the arachidonic acid metabolites produced by the cytochrome P450 (CYP) enzyme. The elevated concentration of 20-HETE was found to be related to the dysfunctions of endothelial and smooth muscle cells, resulting in hypertension or inflammation (Kroetz and Xu, 2005; Lu et al., 2014; Szczuko et al., 2020). The excretion of 20-HETE was found to be negatively correlated with the insulin level. A high level of insulin might result in the dysfunctional synthesis of 20-HETE. This could be caused by the inhibition of phospholipase A2 (Szczuko et al., 2020). The level of 20-HETE fluctuated drastically during late pregnancy. Although an increased level of 20-HETE in the microsomes isolated from placental vessels was observed, the circulating level of 20-HETE in preeclamptic patients showed no significant difference compared with healthy pregnant women (Plenty et al., 2018). The potential effect of 20-HETE on sodium excretion could lead to a reduction in blood pressure in the second and third trimesters of pregnancy (Kikut et al., 2020). Moreover, 20-HETE was shown to have a similar effect as oxytocin in inducing uterine muscle contractions. Our results suggested that an uneventful late pregnancy was characterized by an increase in the levels of long-chain fatty acids in fecal samples. This finding demonstrated that the changes of some crucial fatty acids might have the immunomodulation effects, resulting in the transformation to a proinflammatory immune state during the third trimester, which is important for labor.

The KEGG analysis revealed that the pentose phosphate pathway was enriched in the fecal samples. The PPP is a branch of glycolysis and plays a pivotal role in synthesizing ribonucleotides and nicotinamide adenine dinucleotide phosphate (NADPH). The PPP is closely correlated with high cell proliferation and is essential for the biosynthesis of fatty acids and sterols (Giacomini et al., 2020). A longitudinal cohort study of 232 healthy pregnant women also indicated that the levels of the intermediate product of glycolysis or gluconeogenesis increased, while intermediates involved in the tricarboxylic acid cycle decreased significantly in the second and third trimesters of pregnancy (Wang et al., 2018). In our study, the levels of D-gluconate, which is an important metabolite of the PPP, showed an upward trend during late pregnancy. The increased activation of the pentose phosphate pathway suggested that the absorption and utilization of carbohydrates and the synthesis of other substances by maternal cells increased to meet the demands of normal fetal growth and development. Combined with the findings regarding the biological correlation of the gut microbiota, these findings of our study might indicate the involvement of the underlying mechanism of insulin resistance during late pregnancy, in addition to with the reduction in the aerobic breakdown of glucose.

Furthermore, a decrease in the levels of some essential amino acids and derivatives was detected in both the fecal and plasma samples. KEGG analysis revealed that the pathways of phenylalanine metabolism were enriched during late pregnancy. This result suggested that a large amount of amino acids in the plasma of pregnant women were transported to the fetus and enriched in the placental tissue for further utilization. In addition, the placenta made great use of leucine, valine and isoleucine, some of which produced α-ketoic acid and ammonia under the action of transaminase (Manta-Vogli et al., 2020). Caprylic acid is an eight-carbon straight-chain fatty acid and is also known as octanoic acid. It can be found naturally in numerous foods and breast milk (Lemarié et al., 2018). Caprylic acid was found to have strong antibacterial activity and a positive effect due to changes in intestinal epithelium structure or in the microbiota of the gastrointestinal tract (Świątkiewicz et al., 2020). In our study, the level of caprylic acid presented a downward trend in the plasma at the full term. The decrease of caprylic acid might have been the consequence of the diffusion across membranes to placenta and metabolism by the fetus. It was reported that the placenta expressed the enzymatic machinery required for mitochondrial fatty acid (FA) oxidation. The placental FA oxidation pathway has several physiological effects in pregnancy. Octanoic acid oxidation was found to be 10-fold higher than palmitic acid oxidation in term human placentas undergoing labor (Mendez-Figueroa et al., 2013). The decreased mitochondrial β-oxidation of medium-chain fatty acids might be attributed to the development of fatty liver during late-term pregnancy (Grimbert et al., 1993). The levels of other substances in the plasma or fecal samples gradually increased with gestational age. This finding might have been the result of a complex adaptation for pregnancy. Generally, there were some specific metabolic features observed in the fecal and plasma samples obtained from pregnant women at different stages.

Few studies exist on the differential metabolites and gut microbiota features that are involved in physiological activities during pregnancy. We assumed that the correlations between the gut microbiota and metabolism were altered during late pregnancy and contributed to the onset of delivery. The alterations in fecal metabolites were caused by a combined effect of the microbial community instead of a specific microbe (Figure 4). There was no obvious correlation between the levels of plasma metabolites and differential microbes in our study. The differential microbes of *Proteobacteria* were closely associated with the metabolites involved in the pentose phosphate pathway and fatty acids metabolism. As the microbes associated with the gut microbiota and their metabolites are interconnected, pregnancy might be regulated at the level of the gut microbiota or gut microbiota−metabolite and metabolite−metabolite levels. It is possible that specific microbial or microbiota-metabolite relationships play a vital role in biological pathways during the late pregnancy. Additionally, fecal metabolites might further disturb the level of some specific plasma metabolites, leading to variations in host metabolism. For instance, the increased 20-hydroxyarachidonic acid level in the fecal samples was proportional to the levels of phenylalanine, norleucine and valine in the plasma samples. These essential amino-acids play a crucial role in the metabolic network and help maintain metabolic balance for the growth of the fetus. Notably, fatty acid metabolism was crucial during late pregnancy. As the gestational week progressed, the concentration of metabolites (hydroxyisocaproic acid and caprylic acid) which have anti-inflammatory effects, showed a declining trend. In contrast, the increase in 20-hydroxyarachidonic acid and palmitic acid levels might give rise to inflammation during full-term pregnancy. Our earlier study showed that the levels of IL-1β, IL-2, IL-12, and IFN-γ increased from baseline to labor and that microbes had immunomodulatory effects, which helped in the switch to a pro-inflammatory state in the third trimester (Chen et al., 2019). This current study further revealed that the specific metabolites also presented some vital changes during late pregnancy. To the best of our knowledge, the associations between metabolites and the gut microbiota in the third trimester of normal pregnancy have not been studied. This requires further investigation with larger sample sizes.

There were some limitations in our study. First, the cohorts analyzed in our study were ultimately not large enough to obtain remarkable results. Second, the traditional analysis of microbiome datasets presented many challenges and biases. The generated microbiome datasets were compositional (Gloor et al., 2017). The biases in sequencing and microbial analysis could be caused by various factors, including the collection and preservation of samples, the methods of DNA extraction (Yang et al., 2020), and the choice of PCR primers for amplification (Sipos et al., 2007; Plauzolles et al., 2022). Amplicon sequencing of the 16S rRNA gene performed by utilizing primers binding to highly conserved regions is the most common sequencing approach used to evaluate the diversity of bacterial communities in clinical samples. The amplicon sequencing of the 16S rRNA gene could provide microbial information to the phylum level but failed to identify all the bacteria to the species level (Heravi et al., 2020). The observed gut microbiota might not accurately reflect actual taxon densities. Third, the assessment of relative abundance or correlation analysis might have involved bias (Gloor et al., 2017). The relative abundances of microbes in the microbial community were assessed by 16S rRNA high-throughput sequencing. We could not determine the absolute abundance and actual contributing species in the microbial community. Multiple tools or combined methods are necessary for a comprehensive understanding of the microbiome. Further shotgun metagenomic sequencing analysis that covers the whole genomic content of microorganisms could provide greater specificity and sensitivity at the species level and other biological information. The changes in the microbial community should be studied in the context of the actual clinical phenotypes. Furthermore, metabolism during pregnancy is affected by many factors, such as hormonal changes and diet. Metabolites should be monitored on a more continuous basis to investigate the real dynamic changes in metabolism. The structural identification of metabolites was achieved by using a self-built database. The differential metabolites, such as M795T29 and M415T107_2, should be identified definitively with targeted metabolomics. Moreover, it was exceptionally challenging for us to accurately distinguish host-derived metabolites from microbially generated metabolites. Thus, we focused on the differential microbes at the genus level and their correlations with metabolism in the third trimester of pregnancy.

Despite these limitations, our study indicated that there were significant changes in the composition of the gut microbiota and metabolic phenotypes during the third trimester of pregnancy. Evaluation of the interplay between the gut microbiota and metabolism during the third trimester of pregnancy revealed the possibility of executing some early interventions for pregnancy complications and to provide better prenatal care.

## Conclusion

Overall, the correlation networks of the gut microbiota changed dramatically as pregnancy progressed. The biomarkers of the gut microbiota during late pregnancy were far from satisfying the requirement of improving the precision and specificity of diagnoses. The metabolic network in late pregnancy is individualized and complex. The metabolism of the host and microbiota was influenced by various factors. In the third trimester of pregnancy, the excretion of free amino acids, glycolysis products and some specific fatty acids was affected by microbial activities. There was a distinctive metabolic system in late pregnancy. The identified microbiota and metabolites have promising value in further interventions for pregnancy complications and better prenatal care. Further in-depth study on the relationships of the gut microbiota and related metabolites in pregnancy complications will be necessary.

## Data availability statement

The data presented in the study are deposited in the China Nucleotide Sequence Archive (CNSA: https://db.cngb.org/cnsa) repository, accession code CNP0002979.

## Ethics statement

The studies involving human participants were reviewed and approved by the Ethical Committee of the First Affiliated Hospital of Jinan University (approval number: 2019-011). The patients/participants provided their written informed consent to participate in this study.

## Author contributions

XX and XL conceived and designed the study. XL, HL, and XX collected the samples and clinical data. XX and LL supervised the experiments. XL and RW described the methodology, performed the analysis, interpreted the data, and prepared the manuscript. YL and XC polished the manuscript. XX, LL, and XL approved the final manuscript. All authors agreed to the submission and had full access to the final version of the manuscript.

## Funding

This work was supported by grants from the National Natural Science Foundation of China (Nos. 81771664 and 81871217) and the Science and Technology Program of Guangzhou, China (No. 201904010390).

## Conflict of interest

The authors declare that the research was conducted in the absence of any commercial or financial relationships that could be construed as a potential conflict of interest.

## Publisher’s note

All claims expressed in this article are solely those of the authors and do not necessarily represent those of their affiliated organizations, or those of the publisher, the editors and the reviewers. Any product that may be evaluated in this article, or claim that may be made by its manufacturer, is not guaranteed or endorsed by the publisher.

## Footnotes

^1^ http://www.arb-silva.de/

^2^ https://www.r-project.org/

^3^ https://gephi.org/

^4^ http://www.genome.jp/kegg/

^5^ http://www.metaboanalyst.ca/

^6^ http://metorigin.met-bioinformatics.cn/
