# Supplementary material for: The interplay between the gut microbiota and metabolism during the third trimester of pregnancy
Source: Front Microbiol. 2022 Dec 7;13:1059227. doi: 10.3389/fmicb.2022.1059227 (PMC9768424; doi:10.3389/fmicb.2022.1059227)
Supplement: Supplementary file 1 [file Data_Sheet_1.PDF]

Supplementary Material

1 Supplementary Data

1.1 DNA Extraction and Sequencing

Total genome DNA from samples was extracted using CTAB/SDS method. DNA concentration and purity were monitored on 1% agarose gels. According to the concentration, DNA was diluted to 1ng/µl using sterile water. For each sample, we amplified the V3 and V4 hypervariable regions of the 16S rRNA gene using modified 341F (5'-CCTAYGGGRBGCASCAG-3') / 806R (5'-GGACTACNNGGGTATCTAAT-3') primers. All PCR reactions were carried out in 30µL reactions with 15µL of Phusion® High-Fidelity PCR Master Mix (#M0531S, New England Biolabs, USA); 0.2µM of forward and reverse primers, and about 10 ng template DNA. Thermal cycling consisted of initial denaturation at 98°C for 1 min, followed by 30 cycles of denaturation at 98°C for 10 s, annealing at 50°C for 30 s, and elongation at 72°C for 30 s. Finally, 72°C for 5 min. Mix the same volume of 1X loading buffer (contained SYB green) with PCR products and operate electrophoresis on 2% agarose gel for detection. Samples with bright main strip between 400-500bp were chosen for further experiments. PCR products were mixed in equidensity ratios. Then, mixture PCR products were purified with GeneJET Gel Extraction Kit (K0692, Thermo Scientific, USA). Sequencing libraries were generated using Illumina TruSeq DNA PCR-Free Library Preparation Kit (Illumina, USA) following manufacturer's recommendations and index codes were added. The library quality was assessed on the Qubit@ 2.0 Fluorometer (Thermo Scientific) and Agilent Bioanalyzer 5400 system. At last, the library was sequenced on an Illumina NovaSeq platform PE250 at Novogene (Beijing, China).

1.2 16s rRNA Data Analysis

Paired-end reads were allocated to each sample according to the marked barcodes. The assigned paired-end reads from the original DNA fragments were merged by using FLASH (V1.2.7, http://ccb.jhu.edu/software/FLASH/). The merged raw tags were filtered for quality controlled and developed into clean tags according to QIIME (V1.9.1, http://qiime.org/scripts/split_libraries_fastq.html). Sequences with ≥97% similarity were assigned to the same operational taxonomic unit (OTU) using Uparse (v7.0.1, http://www.drive5.com/uparse/). RDP Classifier version 2.2 was applied to annotate taxonomic information based on the 16S SILVA online database (http://www.arb-silva.de/). Alpha diversity analysis was performed by Mothur version

v.1.30 (http://www.mothur.org/). QIIME calculated both weighted and unweighted unifrac, which were phylogenetic measures of beta diversity.

1.3 Metabolite extraction from fecal and serum samples

Aliquots of the samples were stored at −80°C for use in ultra-high-performance liquid chromatography equipped with quadrupole time-of-flight mass spectrometry (UHPLC-QTOF/MS). The samples were thawed at 4°C. After adding 1,000 μL precooled extract solvent (acetonitrile-methanol-ddH2O, 2:2:1, v/v), the samples were vortexed for 30 seconds. The mixed fecal samples were sonicated for 30 minutes in an ice-water bath. The mixtures were then incubated at -20 °C for 10 minutes and centrifuged at 14000 g, 4 °C for 20 minutes. A quality control sample was acquired by mixing an equal amount of supernatant from each of the samples. The supernatants were dried in a vacuum centrifuge. The dried samples were redissolved in 100 μL of acetonitrile-ddH2O (1:1, v/v) solvent and then centrifuged for 15 min (14000g, 4 °C). The supernatants were prepared for UHPLC−QTOF/MS Analysis. The untargeted metabolic profiling analysis was conducted using an UHPLC system (1290 Infinity LC, Agilent Technologies, Santa Clara, California, USA) coupled with a quadrupole time-of-flight (TOF) mass spectrometer (Triple TOF 6600, AB SCIEX) with electrospray ionization (ESI) in positive and negative ionization modes at Shanghai Applied Protein Technology Co., Ltd. The other steps were performed as described previously(Li et al., 2021).

1.4 Untargeted Metabolomic Relative Quantitative Analyses

The raw mass spectrometry data (.wiff scan files) were converted to .mzXML files using ProteoWizard before being imported into freely available XCMS software. After peak matching, retention time alignment, and peak area extraction, the metabolites were identified by comparing the accuracy of m/z values (<25 ppm) and spectrum diagram were interpreted with a self-built database (Shanghai Applied Protein Technology) established with authentic standards. After normalization to the total peak intensity, the processed data were uploaded into SIMCA-P14.1 (Umetrics, Umea, Sweden) for multivariate analysis, including Pareto-scaled principal component analysis (PCA) and orthogonal partial least-squares discriminant analysis (OPLS-DA). Sevenfold cross-validation and response permutation testing were used to evaluate the robustness of the model. The variable importance in the projection (VIP) value of each variable in the OPLS-DA model was calculated to indicate its contribution to the classification.

2 Supplementary Figures and Tables

2.1 Supplementary Figures

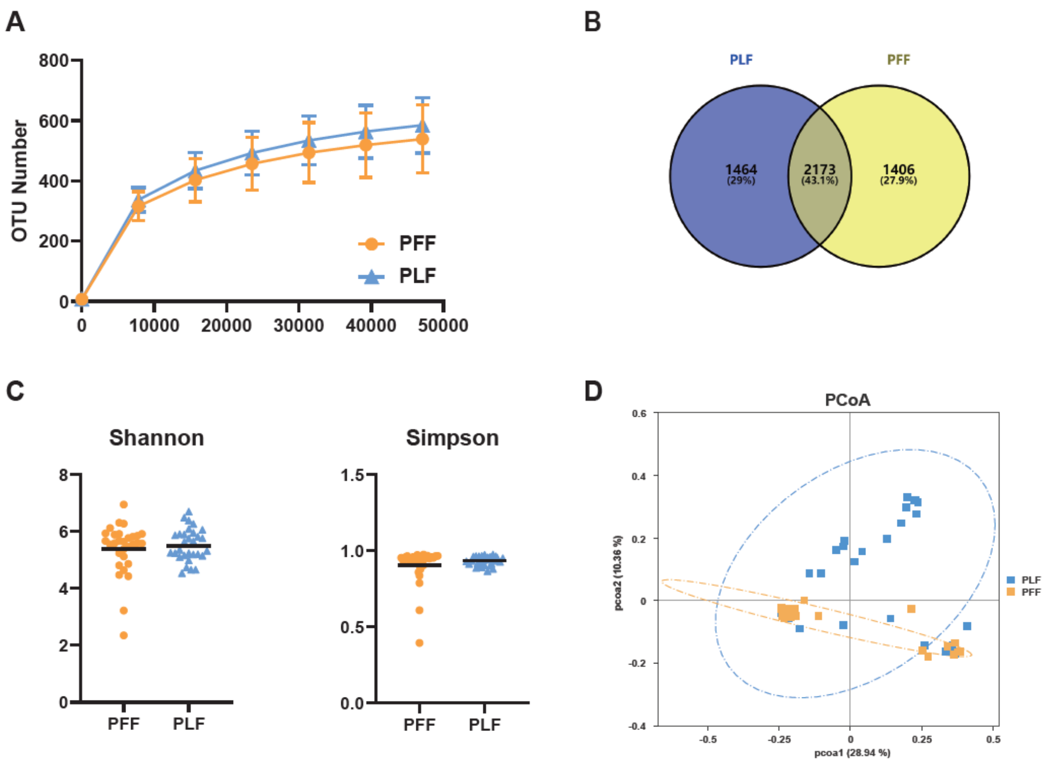

Figure S1. Microbial diversity analysis. (A) The rarefaction curves of data obtained from fecal samples indicated that the OTUs were detected under the sufficient sequencing depth. Means ±95% CI are shown. **(B)** The Venn diagram of the gut microbiota composition at the OTU level. A total of 5043 OTUs at the 97% similarity level were determined. There were 3579 and 3637 OTUs identified in the PFF and PLF groups, respectively. A total of 2173 OTUs were shared among them. **(C)** The alpha-diversity analysis based on the Shannon and Simpson indexes indicated that there was no difference between the two stages. **(D)** The beta-diversity was calculated with an unweighted UniFrac distance matrix. The composition of the gut microbiota was assessed by PCoA. PFF vs. PLF: $P = 0.0021$. PFF, the fecal samples obtained from pregnant women at 30-32 weeks of gestation; PLF, the fecal samples obtained from pregnant women at full term.

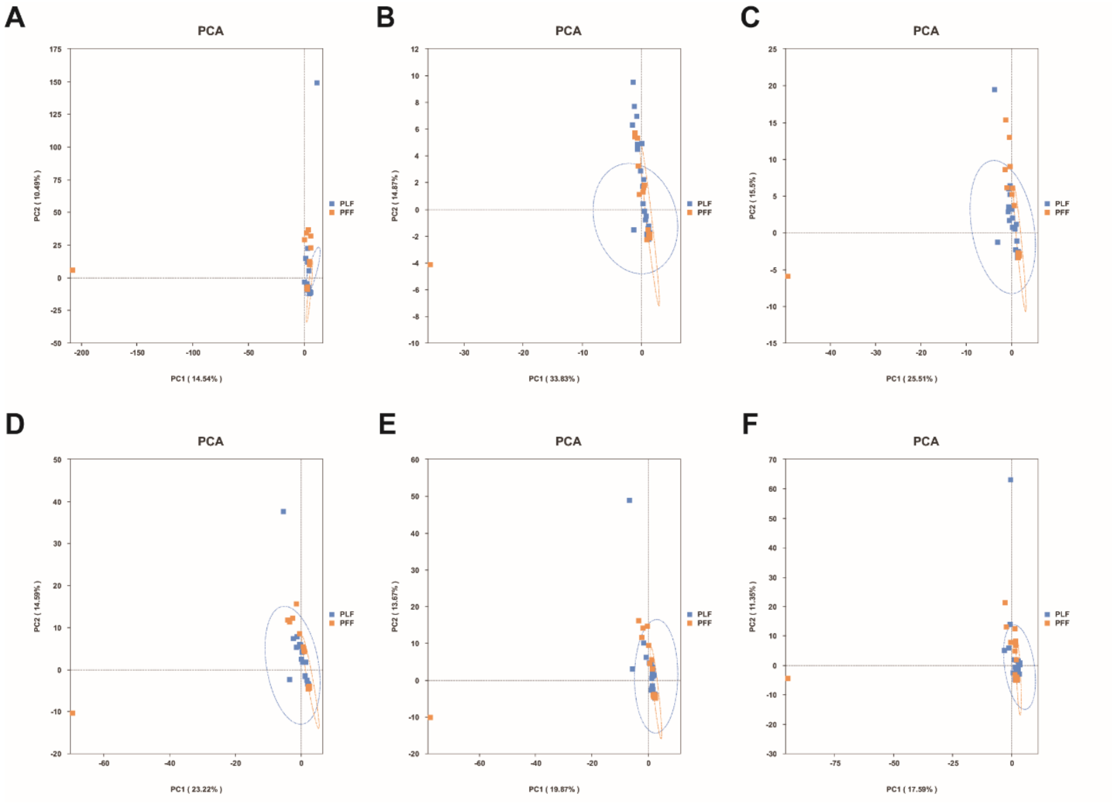

Figure S2. Principal component analysis (PCA) of gut microbiota at different levels. (A) at the OTU level; (B) at the phylum level; (C) at the class level; (D) at the order level; (E) at the family level; (F) at the genus level. Abbreviations: PFF, pregnant women at 30-32 weeks of gestation. PLF, pregnant women at full term.

A

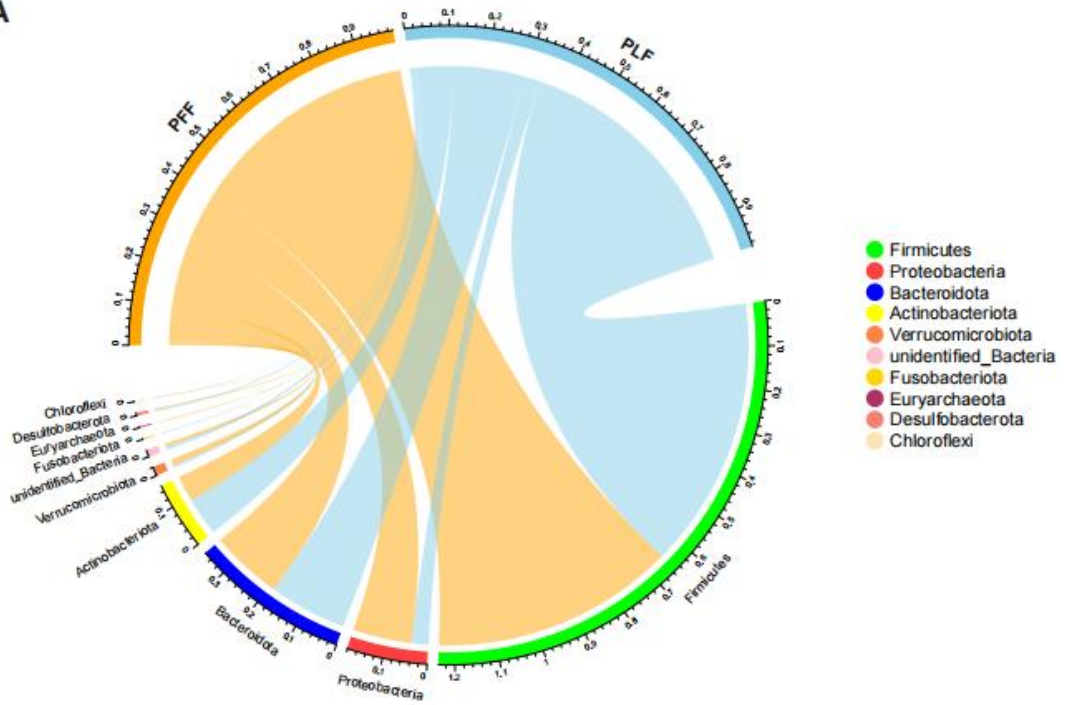

B

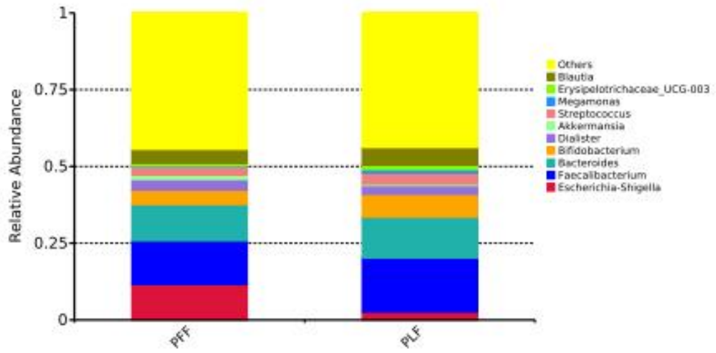

Figure S3. The relative abundance of gut microbiota at different classifications of the two stages. (A) The plot shows the composition of the gut microbiota at the phylum level. The relative abundance of *Proteobacteria* was notably higher at baseline (PFF, 14.14%) than at full term (PLF, 4.05%). **(B)** The plot shows the composition of the gut microbiota at the genus level. *Faecalibacterium*, *Bacteroides* and *Bifidobacterium* showed a remarkable upward trend at the time of full-term pregnancy, whereas *Escherichia-Shigella* (PFF: 11.68%, PLF: 2.58%) showed a downwards trend among the top ten abundant genera. PFF, the fecal samples obtained from pregnant women at 30-32 weeks of gestation; PLF, the fecal samples obtained from pregnant women at full term.

A

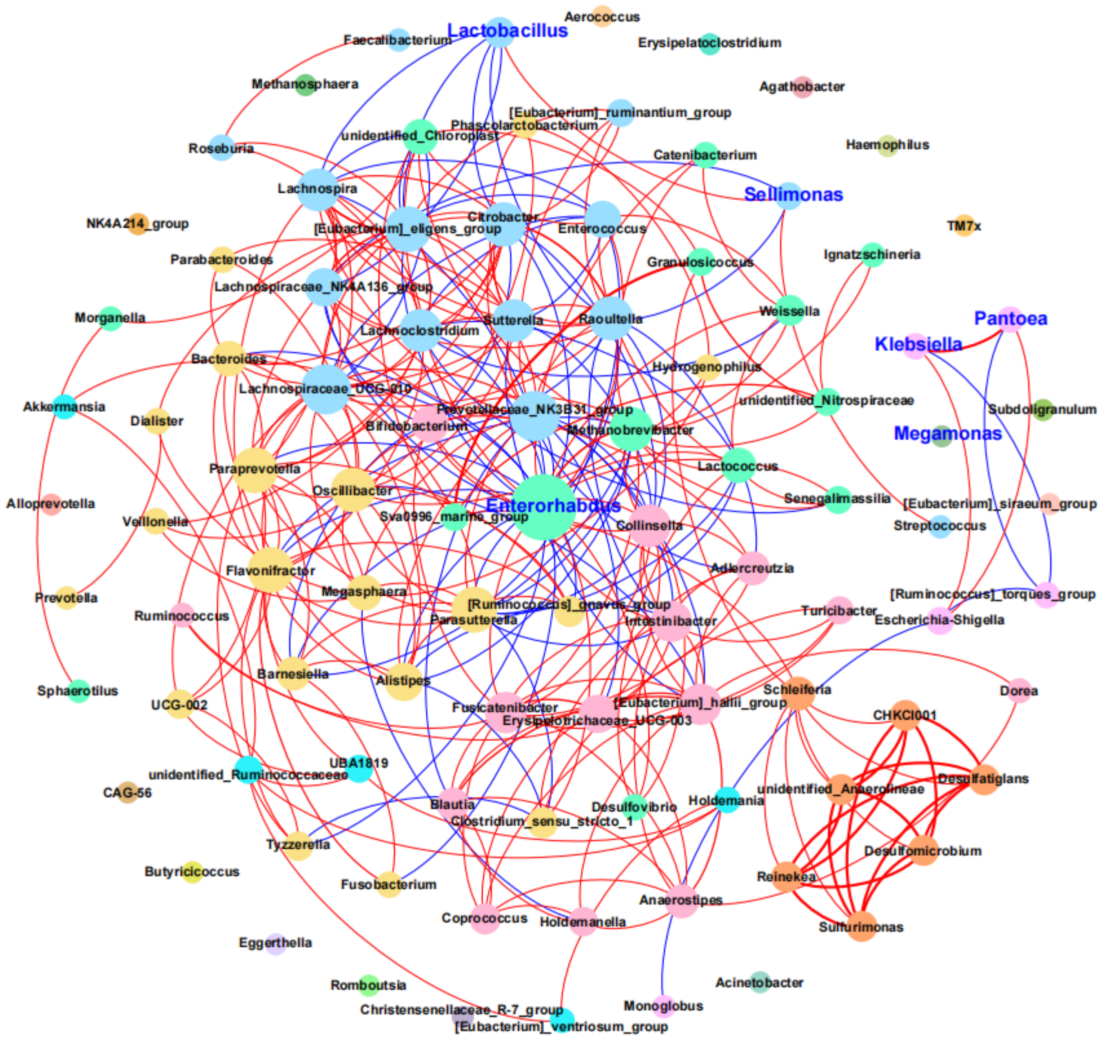
Aerococcus
Lactobacillus
Faecalibacterium
Erysipelatoclostridium
Methanosphaera
Agathobacter
[Eubacterium]_ruminantium_group
Phascolarctobacterium
unidentified_Chloroplast
Roseburia
Catenibacterium
Haemophilus
Lachnospira
Sellimonas
NK4A214_group
Citrobacter
[Eubacterium]_eligens_group
Enterococcus
TM7x
Parabacteroides
Granulosicoccus
Ignatzschineria
Lachnospiraceae_NK4A136_group
Morganella
Sutterella
Raoultella
Weissella
Pantoea
Lachnoclostridium
Klebsiella
Bacteroides
Hydrogenophilus
Lachnospiraceae_UCG-010
unidentified_Nitrospiraceae
Akkermansia
Prevotellaceae_NK3B31_group
Subdoligranulum
Dialister
Bifidobacterium
Methanobrevibacter
Megamonas
Paraprevotella
Lactococcus
Oscillibacter
Alloprevotella
Enterorhabdus
Senegalimassilia
[Eubacterium]_siraeum_group
Sva0996_marine_group
Veillonella
Collinsella
Streptococcus
Flavonifractor
Adlercreutzia
Prevotella
Megasphaera
[Ruminococcus]_torques_group
[Ruminococcus]_gnavus_group
Turicibacter
Ruminococcus
Parasutterella
Intestinibacter
Escherichia-Shigella
Barnesiella
Alistipes
Sphaerotilus
Schleiferia
Dorea
UCG-002
Fusicatenibacter
[Eubacterium]_hallii_group
Erysipelotrichaceae_UCG-003
CHKCI001
UBA1819
unidentified_Ruminococcaceae
Desulfatiglans
CAG-56
unidentified_Anaerolineae
Blautia
Desulfovibrio
Holdemania
Clostridium_sensu_stricto_1
Tyzzerella
Desulfomicrobium
Butyricicoccus
Reinekea
Fusobacterium
Anaerostipes
Coprococcus
Holdemanella
Sulfurimonas
Eggerthella
Romboutsia
Acinetobacter
Christensenellaceae_R-7_group
Monoglobus
[Eubacterium]_ventriosum_group

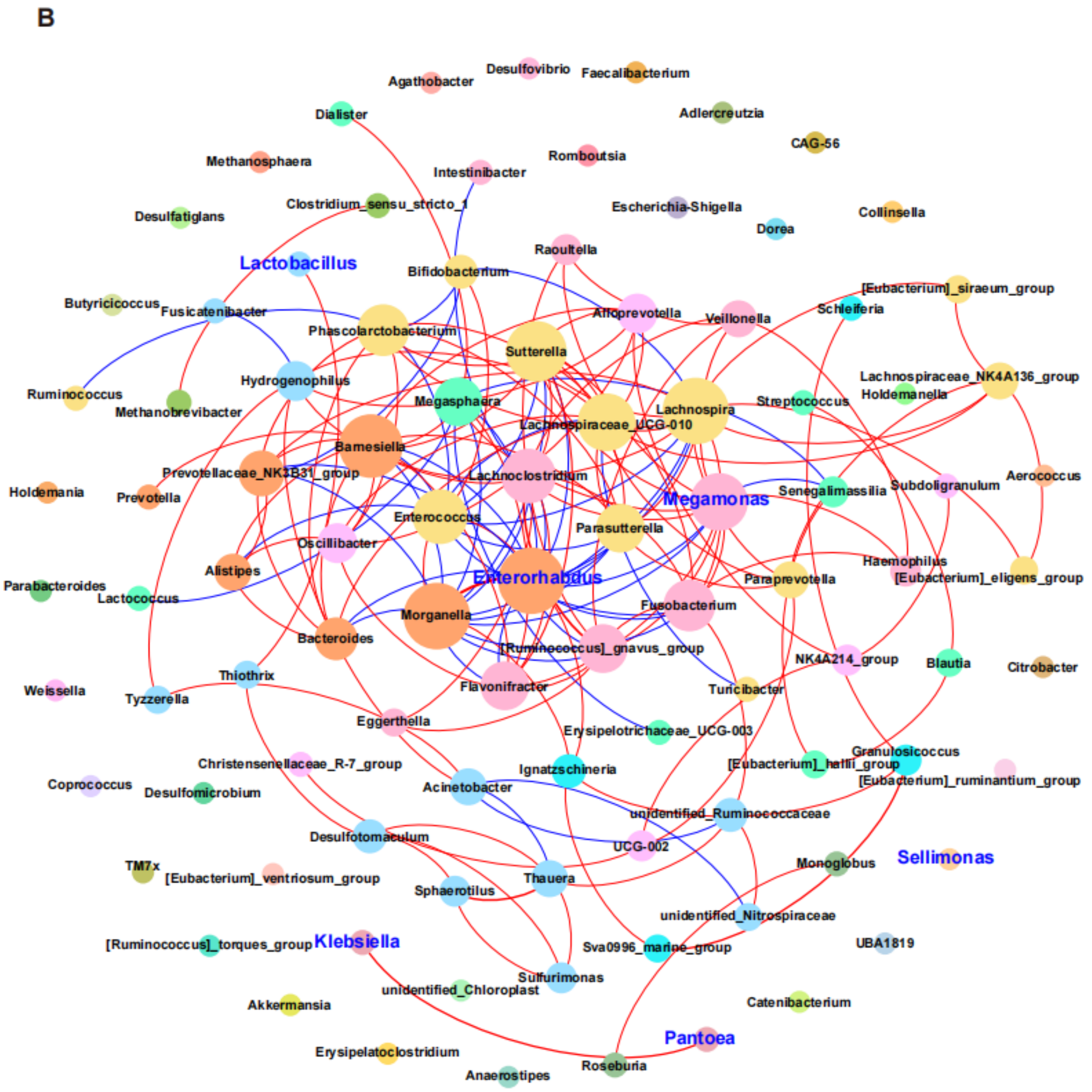

Figure S4. The network of gut microbiota at the genus level in late pregnancy. Each node was annotated with the name. The size of the nodes indicates their degrees in each group. The color of each node represents the modularity. The red and blue lines represent the positive and negative relationships between each node, respectively. **(A)** At 30-32 weeks of gestation, the microbial community was complex and balanced. Each module was tightly connected. **(B)** As the gestational age progressed, some genera switched from one module to another. Many more modules were observed.

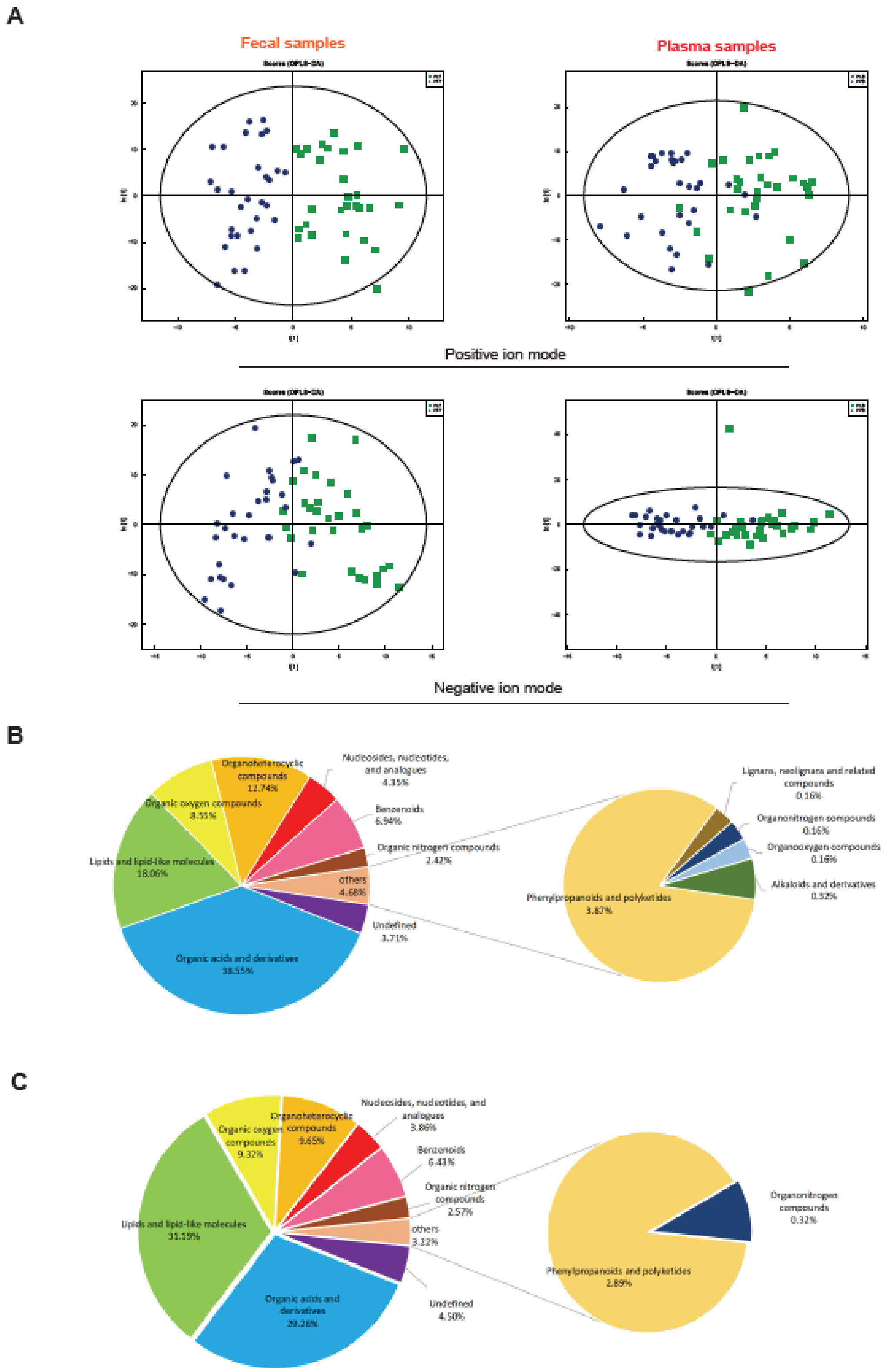

Figure S5. (A) OPLS-DA score plots for fecal and plasma samples obtained from pregnant women at two stages. The results indicated that there was a separation of the pregnant women at different phases.

1) Fecal samples: PFF vs. PLF. In the positive ion mode, R^2X(cum) was 0.106, R^2Y(cum) was 0.774, and Q^2(cum) was -0.794. The R^2X(cum) was 0.152, R^2Y(cum) was 0.647, and Q^2(cum) was -0.306 in the negative ion mode. 2) Plasma samples: PFB vs. PLB. In the positive ion mode, R^2X(cum) was 0.243, R^2Y(cum) was 0.605, and Q^2(cum) was -0.11. The R^2X(cum) was 0.184, R^2Y(cum) was 0.706, and Q^2(cum) was 0.356 in the negative ion mode. **(B)** The compositions of identified fecal metabolites are shown by their classifications. PFF, the fecal samples obtained from pregnant women at 30-32 weeks of gestation; PLF, the fecal samples obtained from pregnant women at full term. **(C)** The compositions of identified plasma metabolites are shown by their classifications. PFB, the plasma samples obtained from pregnant women at 30-32 weeks of gestation; PLB, the plasma samples obtained from pregnant women at full term.

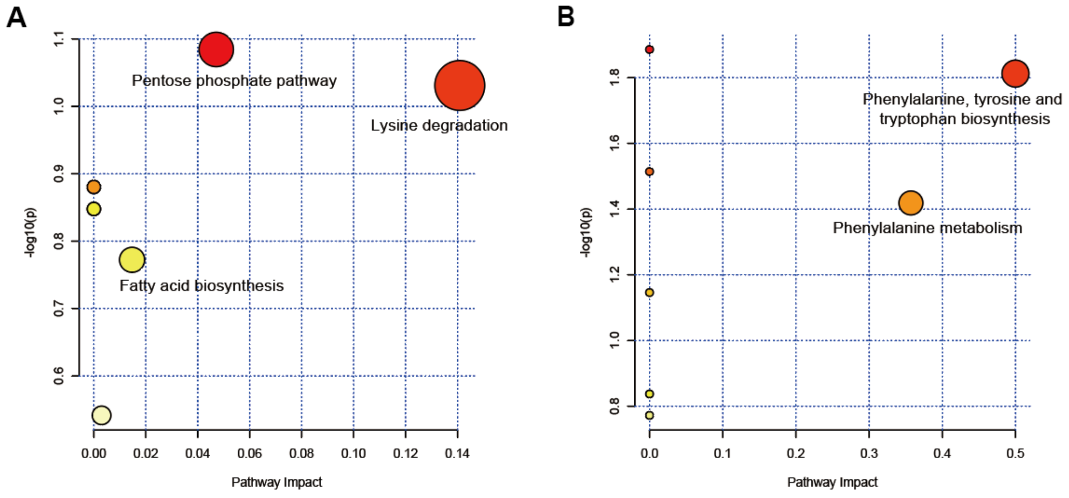

Figure S6. The enriched pathway analyses of differential metabolites. Metabolic pathway enrichment of (A) fecal metabolites and (B) plasma metabolites.

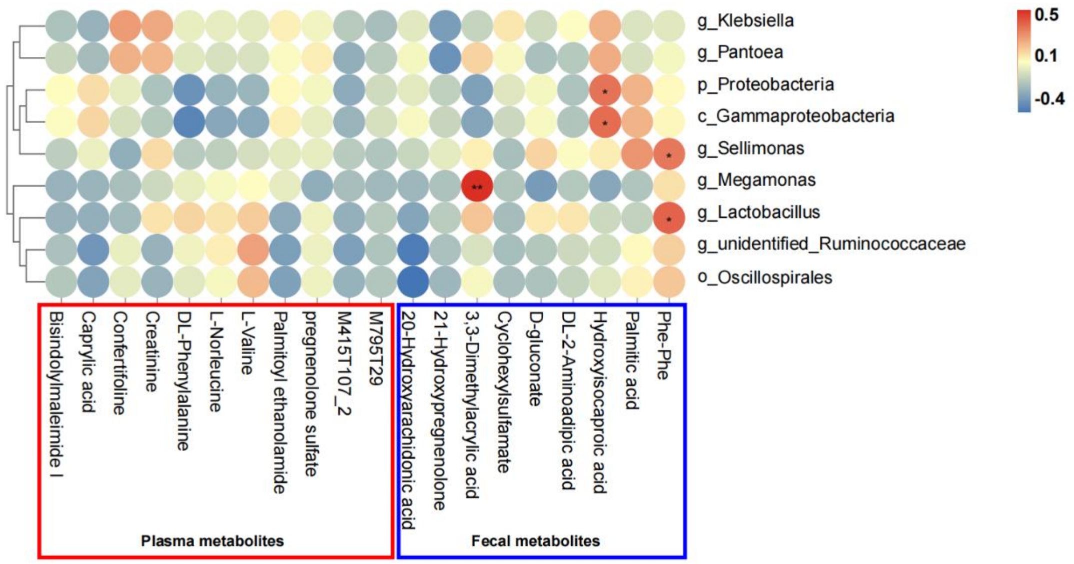

Figure S7. The correlations between fecal microbes, fecal metabolites and plasma metabolites in the full-term pregnancy. *P <0.05; **0.001≤P <0.01.

2.2 Supplementary Tables

Table S1 Clinical characteristics of enrolled participants.

	Participants(n=30)
Age(years)	26.67±2.15
Progestational BMI (kg/m^2)	19.90±1.46
Weight gained during pregnancy(kg)	13.35±4.42
Days of pregnancy(d)	277.00±7.04
Vaginal/Cesarean [a]	25/5
Postpartum bleeding(ml)	359.17±132.46
Infant gender(male/female)	17/13
Infantile birth weight(kg)	3201.67±287.52
Infantile birth body length(cm)	49.60±1.33
Infantile head circumference(cm)	33.50±0.73
One-minute Apgar score of neonates [b]	9

BMI, body mass index; [a] mode of delivery. [b] the median score.

Table S2 The correlations among fecal metabolites in full-term pregnancy

Fecal metabolites	Fecal metabolites	r	*P*-value
Phe-Phe	3,3-Dimethylacrylic acid	0.407	0.025*

r, *Pearson* correlation coefficient; **P* <0.05; **0.001≤*P* <0.01; ****P* <0.001.

Table S3 The correlations among plasma metabolites in full-term pregnancy

Plasma metabolites	Plasma metabolites	r	*P*-value
Caprylic acid	Bisindolylmaleimide I	0.562	0.001**
DL-Phenylalanine	Caprylic acid	-0.426	0.019*
L-Norleucine	Caprylic acid	-0.384	0.036*
L-Norleucine	DL-Phenylalanine	0.922	< 0.001***
L-Valine	Bisindolylmaleimide I	-0.410	0.024*
L-Valine	Caprylic acid	-0.490	0.006**
L-Valine	DL-Phenylalanine	0.845	< 0.001***
L-Valine	L-Norleucine	0.938	< 0.001***
Palmitoyl ethanolamide	L-Norleucine	-0.404	0.027*
pregnenolone sulfate	Bisindolylmaleimide I	0.392	0.032*
M415T107_2	Bisindolylmaleimide I	0.548	0.002**
M415T107_2	DL-Phenylalanine	-0.440	0.015*
M415T107_2	L-Norleucine	-0.465	0.010*
M415T107_2	L-Valine	-0.437	0.016*
M795T29	Bisindolylmaleimide I	0.621	< 0.001***
M795T29	Caprylic acid	0.643	< 0.001***
M795T29	Confertifoline	-0.368	0.046*
M795T29	DL-Phenylalanine	-0.429	0.018*

M795T29	L-Valine	-0.378	0.039*
M795T29	M415T107_2	0.525	0.003**

r, *Pearson* correlation coefficient; *P <0.05; **0.001≤P <0.01; ***P <0.001.

Table S4 The correlations between gut microbes and fecal metabolites in full-term pregnancy

Gut microbiota	Fecal metabolites	r	*P*-value
g_Megamonas	3,3-Dimethylacrylic acid	0.536	0.002**
p_Proteobacteria	Hydroxyisocaproic acid	0.392	0.032*
c_Gammaproteobacteria	Hydroxyisocaproic acid	0.406	0.026*
g_Sellimonas	Phe-Phe	0.380	0.038*
g_Lactobacillus	Phe-Phe	0.425	0.019*

c_:Class; g_:genus; r, *Pearson* correlation coefficient; *P<0.05; **0.001≤P <0.01; ***P <0.001.

Table S5 The correlations between plasma metabolites and fecal metabolites in full-term pregnancy

Plasma metabolites	Fecal metabolites	r	*P*-value
Caprylic acid	Palmitic acid	0.588	< 0.001***
Confertifoline	Cyclohexylsulfamate	0.434	0.017*
DL-Phenylalanine	20-Hydroxyarachidonic acid	-0.396	0.030*
DL-Phenylalanine	Palmitic acid	-0.370	0.044*
L-Norleucine	20-Hydroxyarachidonic acid	-0.395	0.031*
L-Valine	20-Hydroxyarachidonic acid	-0.414	0.023*
pregnenolone sulfate	3,3-Dimethylacrylic acid	-0.366	0.047*

r, *Pearson* correlation coefficient; *P <0.05; **0.001≤P <0.01; ***P <0.001.

Table S6 The origin analysis of differential metabolites from MetOrigin

	Name	Origin	Source	Pathway ID	Pathway
Fecal metabolites	3,3-Dimethylacrylic acid	Food related	HMDB: HMDB0000509	-	-
	20-Hydroxyarachidonic acid	Host	KEGG: C14748	hsa00590	Arachidonic acid metabolism
	Phe-Phe	Unknown	-	-	-
	DL-2-Aminoadipic acid	Co-Metabolism	KEGG: C00956; BIGG: NA, L2aadp	ko00311; ko00300; ko00310	Penicillin and cephalosporin biosynthesis; Lysine biosynthesis; Lysine degradation
	Palmitic acid	Co-Metabolism	KEGG: C00249	ko01040; ko00062; ko00071; ko00061	Biosynthesis of unsaturated fatty acids; Fatty acid elongation; Fatty acid degradation; Fatty acid biosynthesis
	Cyclohexylsulfamate	Drug related	KEGG: C02824	-	-
	Hydroxyisocaproic acid	Microbiota	HMDB: HMDB0000746	-	-
	21-Hydroxypregnenolone	Host	KEGG: C05485	hsa00140	Steroid hormone biosynthesis
	D-gluconate	Co-Metabolism	HMDB: HMDB0000625; KEGG: C00257	ko00030	Pentose phosphate pathway
Plasma metabolites	Palmitoyl ethanolamide	Drug related	KEGG: C16512; DrugBank: DB14043	-	-
	Creatinine	Microbiota	KEGG: C00791	ko00330	Arginine and proline metabolism
	L-Norleucine	Drug related	DrugBank: DB15458, DB04419	-	-
	DL-Phenylalanine	Co-Metabolism	KEGG: C00079	ko00970; ko00400; ko00360; ko00998	Aminoacyl-tRNA biosynthesis; Phenylalanine, tyrosine and tryptophan biosynthesis; Phenylalanine metabolism; Biosynthesis of various secondary metabolites - part 2
	L-Valine	Co-Metabolism	KEGG: C00183	ko00970; ko00311; ko00290; ko00290; ko00280	Aminoacyl-tRNA biosynthesis; Penicillin and cephalosporin biosynthesis; Valine, leucine and isoleucine biosynthesis; Pantothenate and CoA biosynthesis; Valine, leucine and isoleucine degradation
	Bisindolylmaleimide I	Drug related	DrugBank: NA, DB03777	-	-
	M795T29	-	-	-	-
	Caprylic acid	Co-Metabolism	KEGG: C06423	ko00785; ko00061	Lipoic acid metabolism; Fatty acid biosynthesis
	pregnenolone sulfate	Drug related	HMDB: HMDB0000774	-	-
	M415T107_2	-	-	-	-
	Confertifoline	-	-	-	-

The specific source information of metabolites can be classified into six groups, including host (mammals), microbiota (archaea, fungi, bacteria), co-metabolism (shared by both host and microbiota), food (food & plant), drug, and environment (toxins & pollutants) (Yu et al., 2022).

Table S7 The biological correlations between differential fecal metabolites and identified gut microbes from MetOrigin

Fecal metabolites	Gut microbes	Involved pathway (pathway ID)
DL-2-Aminoadipic acid	c_Gammaproteobacteria	Lysine biosynthesis (ko00300)
	g_Klebsiella	Lysine biosynthesis (ko00300)

	g_Lactobacillus	Lysine biosynthesis (ko00300)
Palmitic acid	c_Gammaproteobacteria	Fatty acid biosynthesis (ko00061); Fatty acid degradation (ko00071); Biosynthesis of unsaturated fatty acids (ko01040)
	g_Klebsiella	Fatty acid biosynthesis (ko00061); Fatty acid degradation (ko00071); Biosynthesis of unsaturated fatty acids (ko01040)
	g_Lactobacillus	Biosynthesis of unsaturated fatty acids (ko01040)
	g_Megamonas	Fatty acid biosynthesis (ko00061)
	g_Pantoea	Fatty acid biosynthesis (ko00061); Fatty acid degradation (ko00071); Biosynthesis of unsaturated fatty acids (ko01040)
D-gluconate	c_Gammaproteobacteria	Pentose phosphate pathway (ko00030)
	g_Klebsiella	Pentose phosphate pathway (ko00030)
	g_Lactobacillus	Pentose phosphate pathway (ko00030)
	g_Megamonas	Pentose phosphate pathway (ko00030)
	g_Pantoea	Pentose phosphate pathway (ko00030)

The differential metabolites, related pathways, and potential microbes at different classification levels that might participate in a metabolic reaction biologically were explored. c_: at class level; g_: at genus level.

Reference:

LI, X., GAO, J., SIMAL-GANDARA, J., WANG, X., CAPRIOLI, G., MI, S. & SANG, Y. 2021. Effect of fermentation by Lactobacillus acidophilus CH-2 on the enzymatic browning of pear juice. *LWT,* 147**,** 111489.

YU, G., XU, C., ZHANG, D., JU, F. & NI, Y. 2022. MetOrigin: Discriminating the origins of microbial metabolites for integrative analysis of the gut microbiome and metabolome. 1**,** e10.
